# Antiangiogenic Action of JZL184 on Endothelial Cells via Inhibition of VEGF Expression in Hypoxic Lung Cancer Cells

**DOI:** 10.3390/cells12192332

**Published:** 2023-09-22

**Authors:** Felix Wittig, Liza Pannenberg, Rico Schwarz, Sander Bekeschus, Robert Ramer, Burkhard Hinz

**Affiliations:** 1 Institute of Pharmacology and Toxicology, Rostock University Medical Center, Schillingallee 70, 18057 Rostock, Germany; felix.wittig@med.uni-rostock.de (F.W.); liza.pannenberg@gmail.com (L.P.); rschwarz-arbeit@gmx.de (R.S.); robert.ramer@med.uni-rostock.de (R.R.); 2 ZIK *plasmatis*, Leibniz Institute for Plasma Science and Technology (INP), Felix-Hausdorff-Str. 2, 17489 Greifswald, Germany; sander.bekeschus@inp-greifswald.de

**Keywords:** angiogenesis, monoacylglycerol lipase, JZL184, lung cancer, hypoxia

## Abstract

JZL184, an inhibitor of monoacylglycerol lipase (MAGL) and thus of the degradation of the endocannabinoid 2-arachidonoylglycerol (2-AG), mediates various anticancer effects in preclinical studies. However, studies on the effect of this or other MAGL inhibitors under hypoxia, an important factor in tumor biology and response to cancer therapy, have not yet been performed in cancer cells. In the present study, the impact of the conditioned media (CM) of A549 and H358 lung cancer cells incubated with JZL184 under hypoxic conditions on the angiogenic properties of human umbilical vein endothelial cells (HUVECs) was investigated. Treatment of HUVECs with CM derived from cancer cells cultured for 48 h under hypoxic conditions was associated with a substantial increase in migration and tube formation compared with unconditioned medium, which was inhibited when cancer cells were incubated with JZL184. In this process, JZL184 led to a significant increase in 2-AG levels in both cell lines. Analysis of a panel of proangiogenic factors revealed inhibition of hypoxia-induced vascular endothelial growth factor (VEGF) expression by JZL184. Antiangiogenic and VEGF-lowering effects were also demonstrated for the MAGL inhibitor MJN110. Receptor antagonist experiments suggest partial involvement of the cannabinoid receptors CB_1_ and CB_2_ in the antiangiogenic and VEGF-lowering effects induced by JZL184. The functional importance of VEGF for angiogenesis in the selected system is supported by observations showing inhibition of VEGF receptor 2 (VEGFR2) phosphorylation in HUVECs by CM from hypoxic cancer cells treated with JZL184 or when hypoxic cancer cell-derived CM was spiked with a neutralizing VEGF antibody. On the other hand, JZL184 did not exert a direct effect on VEGFR2 activation induced by recombinant VEGF, so there seems to be no downstream effect on already released VEGF. In conclusion, these results reveal a novel mechanism of antiangiogenic action of JZL184 under conditions of hypoxic tumor–endothelial communication.

## 1. Introduction

In recent years, a number of preclinical reports have been published suggesting inhibition of the enzyme monoacylglycerol lipase (MAGL) as a promising concept to inhibit cancer progression (for review, see [1]). Thus, MAGL inhibitors show antiproliferative [2,3], anti-invasive [2,4], and antimetastatic [4,5] properties in a variety of tumor cells. Furthermore, inhibition of MAGL exhibits beneficial effects on the quality of life of experimental animals, such as reduction of cachexia in a mouse model of bone cancer [5], suppression of lithium chloride-induced vomiting in shrews [6], and reversal of chemotherapy-induced neuropathy in mice [7]. The overall rarely studied associations between the level of MAGL expression in different tumor types and patient outcomes have recently been reviewed [1].

Depending on the experimental model, the mechanism of anticancer effects of MAGL inhibitors is either via inhibition of the degradation of 2-arachidonoylglycerol (2-AG), an endocannabinoid with anticancer properties, or via reduced concentrations of protumorigenic free fatty acids resulting from the inhibition of the degradation of other monoacylglycerides, or both processes [2,8]. With respect to the first mechanism, MAGL inhibitors, along with inhibitors of anandamide (AEA)-degrading fatty acid amidohydrolase (FAAH), are also classified as compounds that enhance endocannabinoid signaling and augment the therapeutic benefits of endocannabinoids (for review, see [1,9]).

One area that remains very poorly investigated concerns the effect of MAGL inhibitors on tumor neovascularization, which is essential for tumor growth and spread. Tumor angiogenesis allows tumors to grow beyond a diameter of 2–3 mm [10]. A major proangiogenic factor in this process represents the vascular endothelial growth factor (VEGF), which is expressed and released under hypoxic conditions and promotes neovascularization via endothelial receptor tyrosine kinases (for review, see [11]). In previously published work, MAGL inhibitor-induced reduction in colon carcinoma xenograft growth was accompanied by decreased VEGF and fibroblast growth factor (FGF)-2 expression [12], suggesting the involvement of antiangiogenic mechanisms of action. In another recent study, parameters of endothelial angiogenesis were reduced by conditioned media (CM) of lung cancer cells previously treated with MAGL inhibitors, clarifying TIMP-1 (tissue inhibitor of metalloproteinases-1) induction in cancer cells as the underlying mechanism [13]. However, mechanistic cell biology studies under hypoxic conditions have not yet been performed with MAGL inhibitors.

In the present study, the effect of the CM of A549 and H358 lung cancer cells incubated under hypoxic conditions with the MAGL inhibitor JZL184 on the angiogenic properties of human umbilical vein endothelial cells (HUVECs) was investigated. JZL184, a piperidine carbamate, is a highly potent and selective MAGL inhibitor that irreversibly inhibits the enzyme by carbamoylation of the active site catalytic serine nucleophile (Ser122), showing IC_50_ values of 2 nM for murine and human MAGL and 25 nM for rat MAGL [14]. In our previous studies, this MAGL inhibitor has already shown anti-invasive and antiangiogenic effects in normoxic lung cancer cells in vitro as well as antimetastatic and tumor-regressive effects in mice subjected to A549 lung cancer metastasis and xenograft models, respectively [4,13]. Here, we present cannabinoid receptor-dependent antiangiogenic properties of JZL184 due to decreased expression of VEGF in hypoxic lung cancer cells, which in turn leads to decreased activation of VEGF receptor 2 (VEGFR2) in HUVECs. Consequently, a novel mechanism of antiangiogenic action of MAGL inhibitors under conditions of hypoxic tumor–endothelial communication was elucidated.

## 2. Materials and Methods

### 2.1. Materials

JZL184 (#Cay13158), AM-251 (#Cay71670), AM-630 (#Cay10006974), 2-AG (#Cay62160), arachidonoyl ethanolamide (AEA; #Cay90050), palmitic acid (PA; #Cay10006627), palmitoyl ethanolamide (PEA; #Cay90350), oleoyl ethanolamide (OEA; #Cay90265), 2-AG-d5 (#Cay362162), and AEA-d8 (#Cay390050) were obtained from Cayman Chemical (Ann Arbour, MI, USA). Recombinant human VEGF-165 (rVEGF, #HZ-1038) and recombinant human serum albumin (rHSA, #HZ-3001) were purchased from Proteintech (Planegg-Martinsried, Germany). Dimethyl sulfoxide (DMSO), glycerin, glycine, sodium chloride (NaCl), sodium hydroxide (NaOH), hydrochloric acid 37% (HCl), Tris hydrocloride (Tris-HCl), and Tris ultrapure were from AppliChem (Darmstadt, Germany). Bortezomib (#sc-217785) was obtained from Santa Cruz Biotechnology (Heidelberg, Germany). β-Mercaptoethanol was purchased from Ferak (Berlin, Germany). *Aqua ad iniectabilia* was bought from Braun Melsungen (Melsungen, Germany). Ethyl acetate and water for chromatography (LC-MS grade) were obtained from Merck (Darmstadt, Germany). Formic acid was purchased from Honeywell Fluka (Seelze, Germany). MJN110 (#SML0872), capsazepine (#C191), aprotinin, hydrogen peroxide solution (H_2_O_2_), luminol, bromophenol blue, methylthiazolyldiphenyl-tetrazolium bromide (MTT), orthovanadate, *p*-coumaric acid, paraformaldehyde (PFA), and phenylmethylsulfonyl fluoride (PMSF) were from Sigma-Aldrich (Taufkirchen, Germany). Methanol and acetonitrile (ACN) were from J. T. Baker (Phillipsburg, NJ, USA). Leupeptin was bought from Biomol (Hamburg, Germany). Acrylamide (Rotiphorese^®^ Gel 30), ammonium peroxydisulphate (APS), crystal violet, *N*,*N*,*N*′,*N*′-tetramethylethylenediamine (TEMED), and Tween^®^ 20 were obtained from Carl Roth (Karlsruhe, Germany). Dulbecco’s Phosphate-Buffered Saline (DPBS) and fetal bovine serum (FBS) were purchased from PAN-Biotech (Aidenbach, Germany). Non-Fat Milk (NFM) powder was obtained from Bio-Rad Laboratories (Munich, Germany). Gibco^TM^ Penicillin-Streptomycin (10,000 U/mL), Gibco^TM^ Trypsin-EDTA, and Gibco^TM^ Trypan Blue Solution were from Thermo Fisher Scientific (Schwerte, Germany).

### 2.2. Cell Culture

The human lung carcinoma cell line A549 was obtained from DSMZ (Deutsche Sammlung von Mikroorganismen und Zellkulturen, Braunschweig, Germany; #ACC-107; RRID:CVCL_0023). NCI-H358 human lung carcinoma cells were purchased from ATCC (Manassas, Virginia, USA; #CRL-5807^TM^; RRID:CVCL_1559). Both cell lines were cultured in Dulbecco’s Modified Eagle Medium (DMEM) with 4.5 g/L glucose and with UltraGlutamine^TM^ I from Lonza Cologne (Cologne, Germany) supplemented with 10% (*v*/*v*) heat-inactivated FBS, 100 U/mL penicillin, and 100 μg/mL streptomycin. Human umbilical vein endothelial cells (HUVECs) were obtained from PromoCell as single donor cryogenic vials (Heidelberg, Germany; #C-12200; RRID:CVCL_2959) and cultured with the Endothelial Cell Growth Medium Kit (#C-22110, designated HUVEC complete medium) from the same company. In addition, 100 U/mL penicillin and 100 μg/mL streptomycin were added to this medium. All three cell lines used were cultured in a humidified incubator at 37 °C and 5% CO_2_. In experiments with HUVECs, the latter were cultivated for a maximum of five weeks. A549 and H358 cells were frozen in large stock at early passages and used within three months following resuscitation.

With the exception of viability assays with HUVECs, all incubations with test compounds were performed in serum-free DMEM after cells were washed with DPBS. Test substances were dissolved in DMSO (JZL184, MJN110, AM-251, AM-630, capsazepine, palmitic acid) or DPBS (rVEGF, rHSA), after which stock solutions were diluted with DPBS. The final solvent concentrations per compound used in the cell incubates were 0.1% (*v*/*v*) DMSO or 0.000025% (*w*/*v*) rHSA. Even when multiple test substances were added to the cells, the final concentration of DMSO in the incubates did not exceed 0.3% (*v*/*v*) DMSO. In all experiments, the incubation media of vehicle- and substance-treated cells contained the same amount of solvent. When experiments were performed under hypoxia, cells were incubated in the humidified Baker Ruskinn InvivO_2_^®^ 400 chamber (I&L Biosystems, Königswinter, Germany; hereafter referred to as hypoxia chamber) at 1% O_2_, 5% CO_2_, and 37 °C. For equilibration, cells were washed and incubated in serum-free medium under normoxic conditions and subsequently placed in the hypoxia chamber for 15 min. The time after equilibration was defined as the onset of hypoxia. Following equilibration, incubation with the respective test substance or vehicle was performed for the indicated time in the hypoxia chamber. In experiments with the receptor antagonists AM-251, AM-630, and capsazepine, A549 or H358 cells were pre-incubated for 30 min under normoxia followed by hypoxia-equilibration.

### 2.3. Generation and Further Use of Conditioned Media from A549 and H358 Lung Cancer Cells

To generate conditioned media (CM) from A549 and H358 cells, they were seeded at a density of 5 × 10^4^ cells per well of a 48-well plate, grown for 16–24 h under normoxic conditions, washed, equilibrated to hypoxia, and treated for 48 h with the indicated test compounds in a hypoxia chamber with a final volume of 300 µL of serum-free DMEM. In addition, for the wells containing the respective vehicle control without cells, 300 µL of serum-free DMEM, referred to as unconditioned medium (UCM), was added. For experiments with AM-251, AM-630, and capsazepine, a 30 min pre-incubation with these compounds under normoxia was performed prior to hypoxia equilibration. After a 48 h incubation period, UCM/CM were collected and centrifuged at 1300× *g* and 4 °C for 5 min.

CM were then used for HUVEC viability, migration, and tube formation analysis (Section 2.4, Section 2.5, Section 2.6), VEGFR2 Western blot analysis (Section 2.8), LEGENDplex™ multiplex analysis (Section 2.12), and ELISA determination of interleukin (IL)-6, IL-8, tumor necrosis factor (TNF) α, and VEGF (Section 2.13). To obtain a higher total volume of UCM/CM, the corresponding media of a treatment group were pooled in some experiments.

To analyze the viability, migration, or tube formation of HUVECs in relation to the tumor cell secretome, reoxygenated UCM/CM of appropriately treated tumor cells were used to resuspend previously harvested, washed, and counted HUVECs therein.

**Figure 1:**
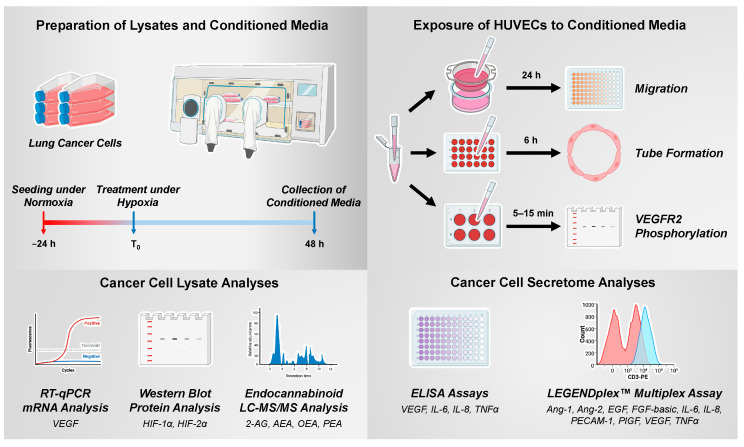
Experimental steps and methods for the generation and use of CM from hypoxic cancer cells and for the analysis of cancer cell lysates (Created with BioRender.com).

The experimental procedures for generating and using CM of hypoxic cancer cells as well as the analyses of their lysates are shown schematically in Figure 1.

### 2.4. Analysis of HUVEC Viability

Viability of HUVEC was determined by a colorimetric assay that detects the cleavage of WST-1 (Roche Diagnostics, Mannheim, Germany), a water-soluble tetrazolium salt, into a soluble formazan dye by metabolically active cells. Absorbance was measured at 450 nm (wavelength correction at 690 nm) using a microplate reader (Infinite F200 Pro Tecan, Tecan Group, Männedorf, Switzerland).

To assess viability after direct exposure to JZL184 and MJN110 (Supplementary Figures S1C and S3C), HUVECs were seeded at a density of 5 × 10^3^ cells per well in 96-well plates and incubated for 24 h with vehicle or the respective test compounds in DMEM supplemented with 2% (*v*/*v*) FBS. For testing the viability of HUVECs after costimulation with rVEGF and JZL184 (Figure 12C), an incubation time of 48 h was chosen. After the experiment-dependent incubation time, the WST-1 reagent was added.

In the case of viability analysis of HUVECs suspended in CM from hypoxic tumor cells (Section 2.3), 100 μL UCM/CM containing 5 × 10^3^ HUVECs were seeded per well of a 96-well plate. In addition, 2% (*v*/*v*) FBS was added to the medium. After an incubation period of 24 h, viability was measured.

In single experiments, the WST-1 assay was also performed directly on Matrigel (Supplementary Figure S2).

### 2.5. Analysis of HUVEC Migration

The effect of test compounds or CM from hypoxic cancer cells on migration of HUVECs was determined by a modified Boyden chamber assay using collagen-coated Falcon^®^ cell culture inserts (#353097; Corning, Corning, NY, USA). In this assay, HUVECs seeded onto the inserts must migrate through a polyethylene terephthalate membrane with 8 µm pores to a chemoattractant in the 24-well companion plate (lower compartment) as previously described [15] with slight modifications. To perform the assay, the lower side of the insert was coated with Corning™ Collagen I (Thermo Fisher Scientific) to improve adherence. DMEM containing 10% (*v*/*v*) FBS, which acts as a chemoattractant, was added to the lower companion plate. After incubation for 24 h in a humidified incubator at 37 °C and 5% CO_2_, the non-migrating HUVECs on the upper surface of the inserts were removed with a cotton swab. Viability, which correlates with the cell number of migrated cells on the bottom side, was quantified using the WST-1 assay.

To examine the direct effects of JZL184 and MJN110 on migration of HUVECs (Supplementary Figures S1A and S3A), 1 × 10^5^ HUVECs were suspended in serum-free DMEM containing vehicle or the indicated concentrations of each test compound.

**Figure 2:**
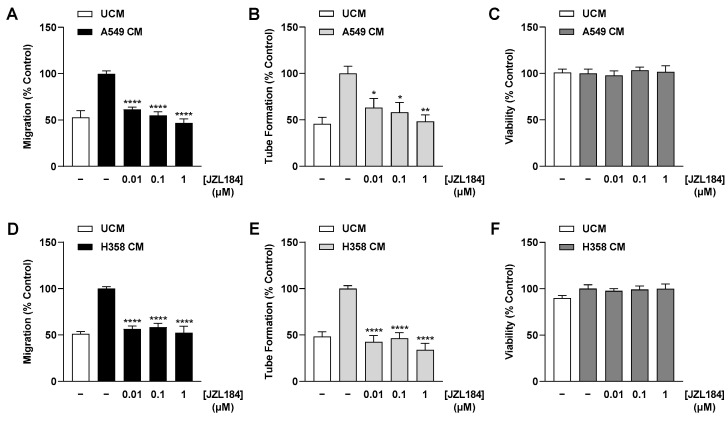
Effect of CM collected from JZL184-treated hypoxic A549 (**A**–**C**) or H358 cells (**D**–**F**) on migration (**A**,**D**), tube formation (**B**,**E**), and viability (**C**,**F**) of HUVECs. Hypoxic A549 and H358 cells, respectively, were treated with vehicle or JZL184 for 48 h, followed by the collection of CM, which were then used to suspend HUVECs. Angiogenic features were determined after incubation of HUVECs with CM from vehicle- or JZL184-treated cells for 6 h (tube formation analysis) or 24 h (migration and viability assay). Percentages shown refer to HUVECs treated with CM derived from vehicle-treated hypoxic cancer cells, each set to 100%. Serum-free DMEM (unconditioned medium, UCM) was included to define the hypoxia effect. Data represent mean ± SEM of *n* = 6 (**B**,**D**,**E**), *n* = 8 (**C**,**F**), or *n* = 9 (**A**). * *p* ≤ 0.05, ** *p* ≤ 0.01, **** *p* ≤ 0.0001 vs. CM from vehicle-treated hypoxic cancer cells; one-way ANOVA with Dunnett’s post hoc test.

In the case of migration analysis of HUVECs in relation to tumor–endothelial communication (Figure 2A,D; Supplementary Figure S3D,G; Figure 4A,C,E,G; Supplementary Figure S4A,C), 1 × 10^5^ HUVECs resuspended in 300 µL CM from A549 or H358 cells (Section 2.3), or UCM, were analyzed.

### 2.6. Analysis of HUVEC Tube Formation

To visualize and quantify the angiogenic potential of test compounds or CM derived from A549 or H358 cells on HUVECs, tube formation assays were performed on 48-well plates coated with Corning^®^ Matrigel^®^ Matrix (#356234; BD Biosciences, Heidelberg, Germany) as previously described [15,16] with slight modifications. Briefly, 48-well plates were coated with 30 µL of ice-cooled Matrigel per well and polymerized at 37 °C for 30 min.

To examine the direct effects of JZL184 and MJN110 (Supplementary Figures S1B and S3B) on tube formation of HUVECs, cells were suspended in serum-free DMEM containing vehicle or the indicated concentrations of each test compound. Then, the corresponding HUVEC suspensions were seeded at a density of 5 × 10^4^ cells per well in a prepared 48-well plate. For analysis of the effects of CM from A549 or H358 cells on HUVEC tube formation (Figure 2B,E; Supplementary Figure S3E,H; Figure 4B,D,F,H; Supplementary Figure S4B,D), 5 × 10^4^ HUVECs were seeded in 200 μL UCM/CM (Section 2.3) per well of a prepared 48-well plate. The subsequent incubation period was 6 h in each case.

Thereafter, the intersections were fixed with 2% (*v*/*v*) PFA, and microscopic images were obtained using the Primovert inverted microscope (Carl Zeiss, Jena, Germany) at 50× magnification for evaluation. Tube formation was quantitatively analyzed in four microscopic fields by counting the number of tube-like structures that formed closed intersections.

### 2.7. Analysis of A549 and H358 Lung Cancer Cell Viability

To investigate the influence of test compounds and siRNAs on tumor cell viability (Supplementary Table S3), A549 and H358 cells were seeded and treated as described in Section 2.3 or Section 2.14. To measure viability, an MTT assay was performed here, which, in contrast to the WST-1 assay, is not sensitive to changes in oxygenation [17]. For this purpose, after an incubation period of 48 h, 60 µL of MTT solution (3 mg/mL in DPBS, 0.5 mg/mL final concentration in incubates) was added and the cells were incubated for another 2 h in the hypoxia chamber. Subsequently, the formed crystals were resolved with DMSO under normoxic conditions and the absorbance was measured at 570 nm (wavelength correction at 690 nm) using a microplate reader.

In the case of palmitic acid, an additional viability assay was performed to indirectly quantify adherent cells and cell death (Supplementary Table S4). For this purpose, cells seeded and treated as described in Section 2.3 were fixed for 30 min with ice-cold absolute ethanol before incubation with crystal violet staining solution (0.1% [*w*/*v*] crystal violet in 10% ethanol) for 30 min. After thoroughly washing off the excess dye, the stained cells were dissolved with 10% acetic acid and the dye intensity was measured at 570 nm with a microplate reader.

### 2.8. Isolation of Total Cellular Protein in Lung Cancer Cells and HUVECs

A549 and H358 cells were seeded in 6-well plates at a density of 2 × 10^5^ per well, except for Figures 9A and 11A, where 2.5 × 10^6^ cells were seeded per Petri dish. Cells were cultured in DMEM containing 10% (*v*/*v*) FBS in a humidified incubator at 37 °C and 5% CO_2_ for 16–24 h. Subsequently, lung cancer cells were washed and the medium was replaced with serum-free medium under normoxic conditions. After hypoxia equilibration for 15 min, cells were treated under hypoxia for fixed incubation periods. If a normoxia control was included, another plate was added with the appropriate incubation times. Cells were then washed with ice-cold DPBS, and after scraping the cells in lysis buffer (2% [*w*/*v*] SDS, 40% [*v*/*v*] H_2_O, 10% [*v*/*v*] glycerol, 50% [*v*/*v*] 125 mM Tris-HCl [pH 6.8]), proteins were immediately denatured at 95 °C under continuous shaking for 10 min. Thereafter, the lysates were centrifuged (20,817× *g*, 4 °C) for 5 min, and the resulting supernatant was collected for further protein analysis.

In the case of p-VEGFR2 and VEGFR2 analysis, HUVECs were seeded in 6-well plates at a density of 3 × 10^5^ per well and cultured for 16–24 h in HUVEC complete medium. HUVECs were then washed and starved for 6 h in serum-free DMEM with or without JZL184 before treatment under normoxic conditions with rVEGF or UCM/CM from A549 or H358 cells (Section 2.3). For neutralization studies, 1 µg/mL VEGF-A antibody (#MAB293; RRID:AB_358222) or 1 µg/mL IgG2B isotype control (#MAB004; RRID:AB_357346) was added to the CM 1 h before at room temperature. Both antibodies were from R&D Systems (Wiesbaden, Germany). After the incubation period, protein isolation was performed as described before.

Protein concentrations were measured using the Pierce™ BCA Protein Assay Kit (Thermo Fisher Scientific). Before samples were stored at −20 °C, 4% (*v*/*v*) β-mercaptoethanol was added.

### 2.9. Isolation of Cytoplasmic and Nuclear Proteins in Lung Cancer Cells

Nuclear and cytoplasmic extraction was performed using NE-PER™ Nuclear and Cytoplasmic Extraction Reagents (#78833; Thermo Fisher Scientific). Briefly, 2.5 × 10^6^ A549 cells were seeded per Petri dish, grown for 16–24 h, and treated as described in Section 2.8. After the incubation period, cells were washed with DPBS supplemented with 1 µM bortezomib and detached with trypsin-EDTA also containing 1 µM bortezomib. Trypsinization was stopped with DMEM supplemented with 10% (*v*/*v*) FBS and 1 µM bortezomib. The cytoplasmic and nuclear pellet was isolated as described in the manufacturer’s instructions. To the CER I and NER solubilization buffers, 10 µg/mL aprotinin, 1 µg/mL leupeptin, 1 mM orthovanadate, 1 mM PMSF, and 10 µM bortezomib were additionally added. Protein concentrations of both fractions were measured using the Pierce™ BCA Protein Assay Kit and samples were stored at −80 °C for further analysis.

### 2.10. Western Blot Analysis of Proteins in Lung Cancer Cells and HUVECs

Equal amounts of protein were separated on an 8% SDS-polyacrylamide gel and transferred to a nitrocellulose membrane, which was then blocked for 1 h in 5% (*w*/*v*) NFM in Tris-buffered saline containing 0.1% (*v*/*v*) Tween^®^ 20 (TBS-T buffer). After washing with TBS-T buffer, membranes were incubated with primary antibodies in 5% (*w*/*v*) NFM overnight at 4 °C. Specific antibodies against VEGFR2 (#2479; RRID:AB_2212507) and p-VEGFR2 (Tyr1175) (#3770; RRID:AB_1642326) were purchased from Cell Signaling Technology (Frankfurt/Main, Germany). HIF-1α (#MA1-516; RRID:AB_325431) was obtained from Thermo Fisher Scientific, HIF-2α (#NB100-122; RRID:AB_10000872) from Novus Biologicals (Wiesbaden-Nordenstadt, Germany), β-actin (#A5441; RRID:AB_476744) from Sigma-Aldrich, and Lamin B1 (#ab16048; RRID:AB_443298) from Abcam (Berlin, Germany). Following washing with TBS-T buffer, membranes were incubated with secondary antibodies coupled to horseradish peroxidase (anti-rabbit antibody: #7074, RRID:AB_2099233 or anti-mouse antibody: #7076; RRID:AB_330924) from Cell Signaling Technology in 5% (*w*/*v*) NFM in TBS-T buffer for 1 h at room temperature. To visualize antibody binding, a chemiluminescent substrate solution (100 mM Tris-HCl [pH 8.5], 1.25 mM luminol; 200 µM *p*-coumaric acid; 0.09% [*v*/*v*] H_2_O_2_) was added, and signal detection was performed using the ChemiDoc XRS gel documentation system from Bio-Rad Laboratories (Munich, Germany). The Precision Plus Protein^TM^ Dual Colour Standard from Bio-Rad Laboratories was used to determine the molecular weight of the bands. Quantification of signal intensity was performed using Quantity One 1-D analysis software (Bio-Rad Laboratories). The signal of a specific protein band was normalized to the signal of the loading control (β-actin for whole-cell lysate and cytoplasmic fraction, lamin B1 for nuclear fraction, and VEGFR2 for p-VEGFR2 analysis).

### 2.11. Quantitative Reverse Transcriptase Polymerase Chain Reaction (RT-qPCR)

A549 and H358 cells were seeded at a density of 2.0 × 10^5^ cells per well in a 6-well plate, cultured for 16–24 h in DMEM containing 10% (*v*/*v*) FBS, washed, and the medium was replaced with serum-free medium under normoxic conditions. After 15 min of hypoxia equilibration, cells were treated under hypoxia for the respective incubation times. Cells were then lysed under hypoxic conditions and total RNA was isolated using the RNeasy Mini Kit from Qiagen (Hilden, Germany). Total RNA concentrations were measured using the NanoDrop™ OneC Microvolume UV-Vis spectrophotometer from Thermo Fisher Scientific to allow the use of equal amounts of RNA for further RT-qPCR. For quantification of VEGF-A, the Applied Biosystems^®^ TaqMan^®^ Gene Expression Assay (Assay ID: Hs00900055_m1; FAM-MGB) and the Applied Biosystems^®^ TaqMan^®^ RNA-to-CT™ 1-Step Kit from Thermo Fisher Scientific were used according to the manufacturer’s instructions. Peptidylprolyl isomerase A (PPIA; Assay ID: Hs999904_m1; VIC-MGB) was used as a housekeeping gene to normalize VEGF-A mRNA levels before comparison with the corresponding vehicle or zero time control.

### 2.12. LEGENDplex™ Multiplex Assay

CM of A549 were obtained as described in Section 2.3 and analyzed for 10 major targets involved in angiogenesis using the LEGENDplex™ multiplex assay (BioLegend, Amsterdam, The Netherlands). The specific kit is the Human Angiogenesis Panel 1 (10-plex) with V-bottom plate (#740698), which allows simultaneous quantification of angiopoietin-1 (Ang-1), angiopoietin-2 (Ang-2), epidermal growth factor (EGF), fibroblast growth factor-basic (FGF-basic), IL-6, IL-8, platelet endothelial cell adhesion molecule-1 (PECAM-1), placental growth factor (PlGF), VEGF, and TNFα. For the experiments, in brief, samples were incubated with capture beads with fluorescent barcodes and coated with monoclonal antibodies against 10 different analytes. After washing, the beads were incubated with phycoerythrin (PE)-conjugated monoclonal antibodies specific for a different epitope of each analyte tested. Following further washing, samples were detected by flow cytometry (CytoFLEX LX; Beckman Coulter, Krefeld, Germany). PE intensities informed absolute analyte amounts by interpolation from 5-log analyte standards measured in parallel.

### 2.13. Determination of VEGF-A, IL-6, IL-8, and TNFα Protein Levels

CM of A549 and H358 cells were obtained as described in Section 2.3. Subsequently, Quantikine^®^ ELISAs from R&D Systems were used for quantification of VEGF-A (VEGF Quantikine ELISA Kit, #DVE00), IL-6 (IL-6 Quantikine ELISA Kit, #D6050), IL-8 (IL-8/CXCL8 Quantikine ELISA Kit, #D8000C) and TNFα (TNF-alpha Quantikine ELISA Kit, #DTA00D). To detect TNFα, 2 mL of CM from a treatment group was pooled and concentrated using the Amicon^®^ Ultra-0.5 Centrifugal Filter Unit (Merck; NMWL: 3 kDa).

### 2.14. Transfection of siRNA in Lung Cancer Cells

Transfection of HIF-1α, HIF-2α, or negative control siRNA was performed using Lipofectamine™ RNAiMAX Reagent (Thermo Fisher Scientific) according to the manufacturer’s instructions provided under “Reverse Transfection” with slight modifications. RNAi-Lipofectamine™ RNAiMAX complexes were prepared in Opti-MEM^®^ I Reduced-Serum Medium (Thermo Fisher Scientific) and contained Lipofectamine™ RNAiMAX reagent and HIF-1α siRNA (Qiagen, #1027418, GeneGlobe ID: SI02664053), HIF-2α siRNA (Qiagen, #1027418, GeneGlobe ID: SI02663038) or negative control siRNA (Qiagen, #1027310). Gently mixed transfection mixtures were added to each well, and after 20 min, A549 or H358 cells in DMEM containing 10% (*v*/*v*) FBS were added (2 × 10^5^ cells per well of a 6-well plate for protein analyses or 5 × 10^4^ cells per well of a 48-well plate for VEGF and viability analyses), yielding a final concentration of 10 nM siRNA or negative control siRNA, respectively. For the use of 0.03 nmol siRNA (single knockdown) as well as 0.06 nmol (double knockdown), 1 µL Lipofectamine™ RNAiMAX reagent was used in each case. After a 24 h incubation period, cells were washed and incubated in serum-free DMEM for the indicated times. Finally, cells were harvested for Western blot analyses or the supernatants were used for VEGF ELISA measurements as described in Section 2.13.

### 2.15. LC-MS/MS Analysis of Endocannabinoids and Endocannabinoid-like Substances in A549 and H358 Lung Cancer Cells

A total of 2.5 × 10^6^ A549 or H358 cells were seeded per Petri dish, grown for 16–24 h, and processed as described in Section 2.8. After each incubation period, cells were washed with DPBS and detached with trypsin-EDTA, and the detached cells from three Petri dishes were combined in a Falcon tube. Cells were harvested as pellets after 5 min centrifugation (173× *g*, 4 °C). The cell pellet was washed with DPBS, recollected after 5 min centrifugation (173× *g*, 4 °C), and stored at −80 °C for at least one day. For the analysis of endocannabinoids (2-AG, AEA) and endocannabinoid-like substances (OEA, PEA), the thawed pellet containing approximately 7.5 × 10^6^ cells was used. The purification and mass spectrometry method was adapted according to previous work of our laboratory [4,18]. The pellets were resuspended in 1 mL of 100 mM Tris-HCl buffer pH 7.4. For subsequent normalization to the appropriate protein levels, 5 µL was taken for protein determination. Extraction was then performed twice with ethyl acetate. Supernatants were concentrated to dryness in a vacuum concentrator (SpeedVac SPD130DLX, Thermo Fisher Scientific, Asheville, NC, USA) and thereafter resuspended in 100 µL of 60% ACN/H_2_O containing 0.2% [*v*/*v*] formic acid. AEA-d8 was added to the samples as an internal standard for AEA, OEA, and PEA and 2-AG-d5 for 2-AG, each at a final concentration of 10 ng/mL. Several standard solutions (for AEA, OEA, and PEA: 0.025, 0.05, 0.1, 0.2, 0.5, 0.75, 1, 2, and 10 ng/mL; for 2-AG: 0.125, 0.25, 0.5, 1, 2.5, 3.75, 5, 10, and 20 ng/mL) were used for calibration. 2-AG was supplied by the producer as a mixture of 2-AG and 1-AG (9:1). Therefore, the stated concentrations refer to nine parts 2-AG and one part 1-AG. A sample volume of 50 µL was injected and separated using a Prominence LC-20AD system (Shimadzu, Duisburg, Germany) equipped with a Multospher 120 C18 AQ column 125 × 2 mm, 5 µm particle size and a guard column (20 mm × 3 mm, 5 µm particle size), both from CS-Chromatographie Service (Langerwehe, Germany). H_2_O was chosen as mobile phase A and ACN as mobile phase B, both containing 0.2% (*v*/*v*) formic acid. The flow rate was 0.3 mL/min. After holding the starting gradient for 3 min, the gradient was increased from 60% B to 100% B in 10 min. 100% B was held for 7 min and then immediately decreased to 60% B to equilibrate the column for 14 min. The oven temperature was set at 40 °C. An LCMS-8050 triple quadrupole mass spectrometer (Shimadzu) was used for the quantification of 2-AG, AEA, OEA, and PEA in cell samples. All mass spectrometric data for positive electrospray ionization and precursor ions with corresponding fragment ions are presented in Supplementary Tables S1 and S2. Argon was used as the gas for collision-induced dissociation (CID). The values shown correspond to the measured concentrations of the analytes normalized to the cellular protein concentrations determined using the Pierce™ BCA Protein Assay Kit.

### 2.16. Statistics

All statistical analyses were performed using GraphPad Prism 9.4.1 (GraphPad Software, San Diego, CA, USA). Comparisons between two groups were carried out using Student’s unpaired two-tailed *t*-test. Comparisons between more than two groups were conducted using one-way ANOVA with Dunnett’s post hoc test when all conditions were compared with vehicle control or using Bonferroni’s post hoc test for selected group comparisons. In some cases (Figures 5, 6A,B and 9), statistical analysis was omitted for content or experimental reasons.

## 3. Results

### 3.1. CM of Hypoxic Lung Cancer Cells Treated with the MAGL Inhibitor JZL184 Inhibit HUVEC Migration and Tube Formation

As recently shown, JZL184 does not directly inhibit the angiogenic properties of HUVECs [13]. Indeed, we were able to confirm this inability in the present study with another HUVEC donor, in which JZL184 even led to a significant increase in migration (Supplementary Figure S1). However, a different pattern was observed when there was a prior interaction with tumor cells. To this end, we generated CM from A549 and H358 lung cancer cells incubated for 48 h under hypoxic conditions with vehicle and increasing JZL184 concentrations, respectively. Under these conditions, JZL184 showed no toxic effect at the tested concentrations compared with vehicle-treated hypoxic A549 and H358 cells (Supplementary Table S3). CM were then used to suspend HUVECs and subsequently to perform appropriate angiogenesis assays. When comparing UCM and CM from hypoxic cancer cells, hypoxia resulted in an increase in HUVEC migration (Figure 2A,D) and tube formation (Figure 2B,E), while HUVEC viability remained largely unchanged (Figure 2C,F). More importantly, inhibition of both hypoxia-induced angiogenic parameters was detected when CM were used from A549 (Figure 2A,B) and H358 cells (Figure 2D,E) previously treated with JZL184. The corresponding effects followed a concentration dependence, with complete inhibitions to UCM levels registered at a JZL184 concentration as low as 0.01 µM when CM of H358 cells were used (Figure 2D,E). An additional control experiment revealed no toxic effect of CM of A549 and H358 cells treated with JZL184 on HUVECs under the incubation conditions of the tube formation assay (Supplementary Figure S2).

Inhibitory effects on migration and tube formation were also detected for the MAGL inhibitor MJN110 in both lung cancer cell lines (Supplementary Figure S3D,E,G,H), whereby MJN110 per se did not show corresponding inhibition upon direct incubation of HUVECs (Supplementary Figure S3A,B). As shown with JZL184, MJN110 also did not cause significant toxic effects on hypoxic A549 and H358 cells at 48 h exposure (Supplementary Table S3).

### 3.2. JZL184 Leads to a Selective Increase of the Endocannabinoid 2-AG in A549 and H358 Cells

**Figure 3:**
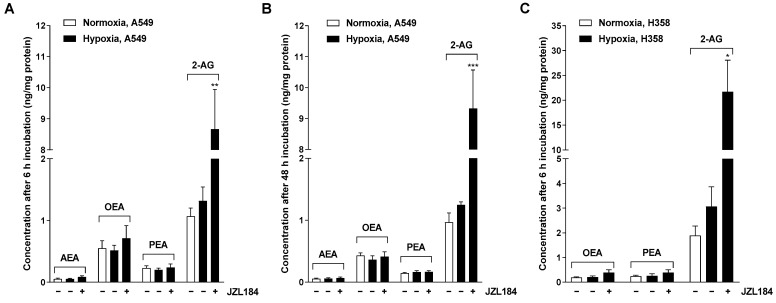
Effect of JZL184 on the levels of endocannabinoids AEA and 2-AG and endocannabinoid-like substances OEA and PEA in hypoxic A549 (**A**,**B**) and H358 cells (**C**). A549 cells were incubated for 6 h (**A**) or 48 h (**B**) and H358 cells were incubated for 6 h (**C**) with vehicle or JZL184 (0.1 µM) under hypoxic conditions. To define the hypoxia effect, vehicle-treated normoxic cancer cells were included in the experiment. Data represent mean concentrations ± SEM of *n* = 4 (**A**,**B**) or *n* = 3 (**C**). * *p* ≤ 0.05, ** *p* ≤ 0.01, *** *p* ≤ 0.001 vs. vehicle-treated hypoxic cancer cells; Student’s unpaired two-tailed *t*-test.

To ensure that the observed effects of JZL184 were indeed accompanied by an increase in the MAGL substrate 2-AG, the endocannabinoids 2-AG and AEA, as well as the endocannabinoid-like substances OEA and PEA, were determined in hypoxic A549 and H358 cells after respective incubation with JZL184. A selectively significant upregulation of 2-AG by JZL184 was confirmed in both cell lines (Figure 3), with AEA, OEA, and PEA also quantified in A549 cells, whereas only OEA and PEA were quantifiable in H358 cells. Interestingly, hypoxia tended to increase 2-AG concentration compared with normoxic cells, although no robust conclusions can be drawn here because of the high variability as well as the different growth conditions of normoxic and hypoxic cells. Finally, as expected, an increase in 2-AG levels was also detected for MJN110 in A549 cells, here with a concomitant increase in OEA and PEA levels, although this was less pronounced compared with 2-AG (Supplementary Figure S3J).

### 3.3. The Antiangiogenic Effect of JZL184 Is Partly Related to Cannabinoid Receptor Activation

**Figure 4:**
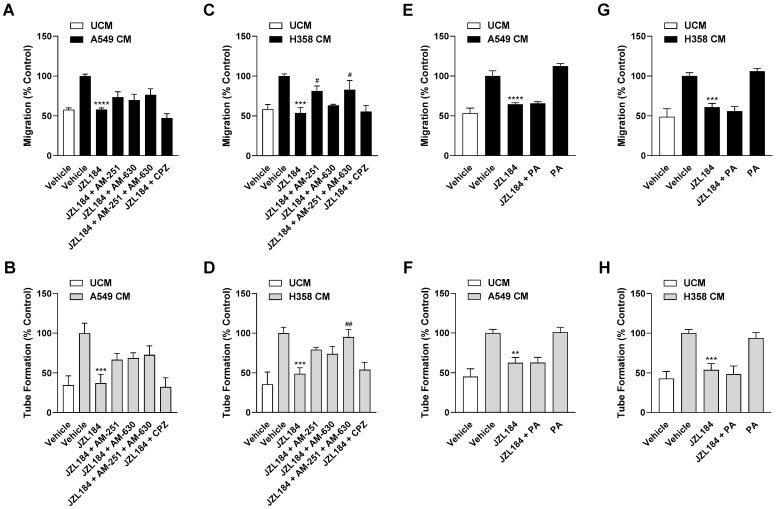
Evaluation of the possible role of the endocannabinoid system and free fatty acid modulation in the antiangiogenic effect of JZL184 on HUVECs. To examine corresponding potential effects, the effect of AM-251 (CB_1_ receptor antagonist), AM-630 (CB_2_ receptor antagonist), capsazepine (CPZ, TRPV1 antagonist), and palmitic acid (PA) on the antiangiogenic effect of CM from hypoxic A549 or H358 cells treated with 0.1 µM JZL184 was tested. Hypoxic cancer cells were incubated with vehicle or JZL184 for 48 h before CM were collected. In (**A**–**D**), cells were pre-incubated for 30 min with the respective receptor antagonist (all tested at a final concentration of 1 µM) under normoxic conditions, while in (**E**–**H**) PA (10 µM) was added to the hypoxic cells concomitantly with JZL184. Migration (**A**,**C**,**E**,**G**) and tube formation (**B**,**D**,**F**,**H**) of HUVECs were determined after incubation with CM of cancer cells for 24 h (migration) or 6 h (tube formation). All percentage values shown refer to HUVECs treated with CM derived from vehicle-treated hypoxic cancer cells, each set to 100%. Serum-free DMEM (unconditioned medium, UCM) was included to define the hypoxia effect. Data represent mean ± SEM of *n* = 6 (**A**–**E**,**G**) or *n* = 9 (**F**,**H**). ** *p* ≤ 0.01, *** *p* ≤ 0.001, **** *p* ≤ 0.0001 vs. CM from vehicle-treated hypoxic cells; # *p* ≤ 0.05, ## *p* ≤ 0.01 vs. CM from JZL184-treated hypoxic cells; one-way ANOVA with Bonferroni´s post hoc test.

To provide further evidence for the endocannabinoid-dependent effect of JZL184 on angiogenesis, interference by receptor antagonists against CB_1_ (AM-251), CB_2_ (AM-630), and TRPV1 (capsazepine) was examined. For this purpose, concentrations that have been shown to be effective in antagonizing cannabinoid effects in different cell cultures [4,15,19,20,21] were used. Noteworthy, for 2-AG, which is inhibited in its degradation by JZL184, activating effects at the CB_1_ and CB_2_ receptor (for review, see [22]) as well as at TRPV1 [23] have been demonstrated in the past. Analysis of the CM of A549 cells revealed partial abrogation of the inhibitory effect of JZL184 on HUVEC migration and tube formation by AM-251 and AM-630, but not by capsazepine (Figure 4A,B). A similar pattern was observed when the CM of H358 cells were analyzed, with AM-251 leading to significant inhibition of migration and the combination of AM-251 and AM-630 causing significant and complete inhibition of the antiangiogenic effect of JZL184 at the level of tube formation (Figure 4C,D). In both cell lines, the antagonists used per se had no appreciable effect on migration and tube formation (Supplementary Figure S4A–D). The effect of the antagonists alone or in combination with JZL184 on the viability of hypoxic A549 and H358 cells was determined under incubation conditions comparable to the collection of CM and is shown in Supplementary Table S3.

Considering that a reduced synthesis of protumorigenic fatty acids can also be registered as a consequence of MAGL inhibition [2,8], a possible decrease in free fatty acids might also be relevant as a cause for the observed antiangiogenic effects of JZL184. To address this, further migration and tube formation assays were performed using palmitic acid with the aim of add-back. However, as shown in Figure 4E–H, the addition of palmitic acid did not abolish the inhibition of migration or tube formation by CM from hypoxic A549 and H358 cells treated with JZL184. The palmitic acid concentration used here resulted in a decrease in cancer cell viability of approximately 20% in the MTT assay targeting metabolic activity (Supplementary Table S3). However, this toxicity could not be confirmed in the crystal violet assay, which focuses on the number of living adherent cells (Supplementary Table S4).

### 3.4. JZL184 Inhibits Hypoxia-Induced VEGF Expression in A549 and H358 Cells

**Figure 5:**
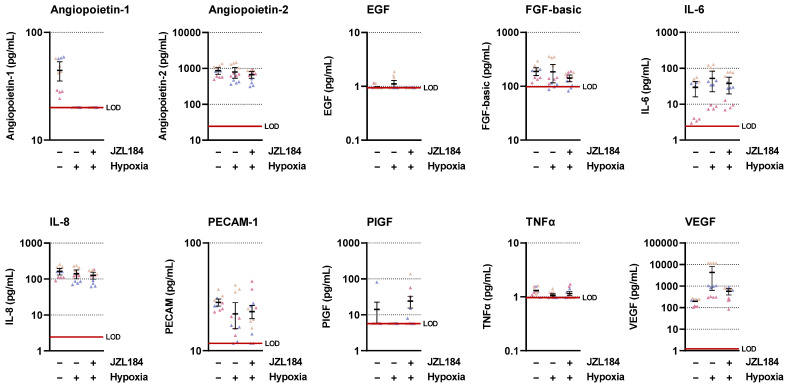
Concentrations of various angiogenic mediators in the CM of vehicle- or JZL184-treated hypoxic A549 cells compared with vehicle-treated normoxic cells. The CM used were from hypoxic A549 cells previously incubated for 48 h with vehicle or JZL184 (0.1 µM). A vehicle-treated normoxic cell group was included to show a potential hypoxia effect. Angiogenic mediators were determined in cell culture supernatants using the LEGENDplex™ multiplex assay. Each independent experiment was measured in four technical replicates (triangles of the same color). The horizontal thick black lines represent the mean values, the vertical ones the SEM of *n* = 3. The horizontal thick red line represents the lower limit of detection (LOD) of the respective analyte. For a better overview of the respective effects, the y-axes are shown logarithmically.

To find a possible mediator for the antiangiogenic effect of JZL184, the regulation of various proangiogenic factors in hypoxic A549 was recorded using LEGENDplex™ multiplex assay (Figure 5). This revealed hypoxia-induced upregulation of VEGF with corresponding inhibition by JZL184. Other proangiogenic factors were either not induced by hypoxia (angiopoietin-2, IL-8, PECAM-1), were within the lower limit of detection with respect to control levels from normoxic or hypoxic cells (angiopoietin-1, EGF, PIGF, TNFα), or showed hypoxia-induced stimulation that was not (IL-6) or not sufficiently (FGF-basic) inhibited by JZL184. Re-examination of the factors IL-6, IL-8, and TNFα by ELISA measurement also confirmed that JZL184 had no effect on the secretion of these factors in A549 cells (Supplementary Figure S5).

**Figure 6:**
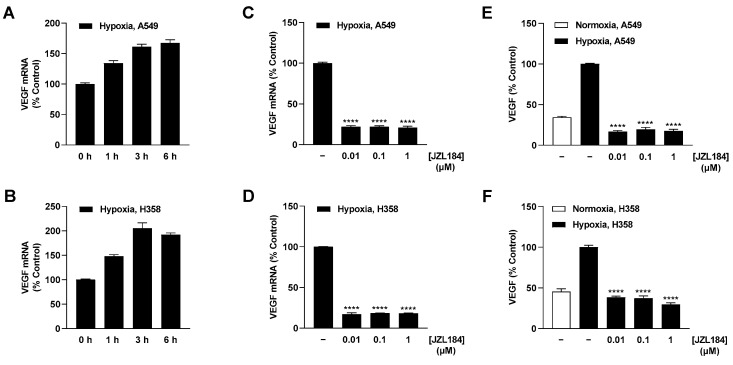
Effect of JZL184 on VEGF mRNA and protein expression in hypoxic A549 and H358 cells. Following the onset of hypoxia, A549 and H358 cells were incubated for the indicated times (**A**,**B**) or with vehicle or JZL184 for 6 h (**C**,**D**) or 48 h (**E**,**F**). Percentage values given refer to zero time (**A**,**B**) or to vehicle-treated hypoxic A549 and H358 cells (**C**–**F**), each set to 100%. For determination of protein (**E**,**F**), a vehicle-treated normoxic cell group was included to define the hypoxia effect. Data represent mean ± SEM of *n* = 6 (**A**–**D**), *n* = 8 (**E**), or *n* = 12 (**F**). **** *p* ≤ 0.0001 vs. vehicle-treated hypoxic cells 6 h (**C**,**D**) or 48 h (**E**,**F**); one-way ANOVA with Dunnett´s post hoc test.

Further analysis of hypoxia-induced VEGF regulation revealed upregulation of this proangiogenic factor at the mRNA level in A549 and H358 cells, which became evident after 1 h (Figure 6A,B). Treatment of tumor cells with JZL184 at the lowest tested concentration of 0.01 µM resulted in approximately 80% inhibition of VEGF mRNA formation (Figure 6C,D) and complete blockade of the corresponding VEGF protein synthesis relative to normoxic levels (Figure 6E,F). Interestingly, the inhibition was a plateau effect since, with the exception of VEGF protein formation in H358 cells, no stronger effects were obtained by JZL184 concentrations of 0.1 or 1 µM. To rule out a possible off-target effect, MJN110 was also used to test the effect of another MAGL inhibitor at this level, which likewise resulted in a profound inhibition of hypoxia-induced VEGF protein formation in both A549 and H358 cells (Supplementary Figure S3K,L).

### 3.5. Inhibition of Hypoxia-Induced VEGF Formation by JZL184 Involves Cannabinoid Receptor-Dependent Events

**Figure 7:**
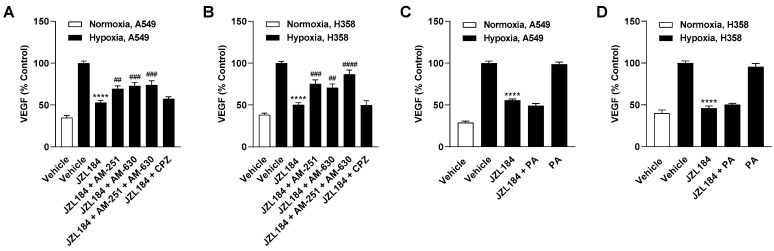
Effect of AM-251 (CB_1_ receptor antagonist), AM-630 (CB_2_ receptor antagonist), capsazepine (CPZ, TRPV1 antagonist), and palmitic acid (PA) on the inhibitory effect of JZL184 (0.1 µM) on VEGF release by hypoxic A549 (**A**) or H358 cells (**B**). Cancer cells were incubated with vehicle or JZL184 for 48 h before CM were collected. In (**A**,**B**), cells were pre-incubated for 30 min with the respective receptor antagonist (all tested at a final concentration of 1 µM) under normoxic conditions, while in (**C**,**D**) PA (10 µM) was added to the hypoxic cells concomitantly with JZL184. VEGF protein concentration was determined in cell culture supernatants by ELISA. All percentage values given refer to vehicle-treated hypoxic A549 or H358 cells, each set to 100%. A vehicle-treated normoxic cell group was included to define the hypoxia effect. Data represent mean ± SEM of *n* = 12 (**A**) or *n* = 8 (**B**–**D**). **** *p* ≤ 0.0001 vs. vehicle-treated hypoxic cells; ## *p* ≤ 0.01, ### *p* ≤ 0.001, #### *p* ≤ 0.0001 vs. JZL184-treated hypoxic cells; one-way ANOVA with Bonferroni´s post hoc test.

To demonstrate a possible involvement of endocannabinoids in VEGF inhibition mediated by JZL184 in hypoxic A549 and H358 cells, experiments with receptor antagonists were also performed. Consistent with the characterization of the antiangiogenic effect of JZL184 (Figure 4A–D), partial inhibition of JZL184-induced VEGF reduction was found in the presence of antagonists against CB_1_ (AM-251), CB_2_ (AM-630), or the combination of both antagonists (Figure 7A,B). Again, the TRPV1 antagonist capsazepine proved to be without effect. Furthermore, an intrinsic stimulatory effect of the receptor antagonists on hypoxia-induced VEGF secretion could be excluded in control experiments (Supplementary Figure S4E,F).

Add-back experiments with palmitic acid were also performed here to compensate for the reduced availability of free fatty acids as a result of MAGL inhibition. As in the angiogenesis assays above, the addition of palmitic acid did not abrogate or attenuate the inhibition of hypoxia-induced VEGF release in A549 and H358 cells mediated by JZL184 (Figure 7C,D).

### 3.6. VEGF Released from Hypoxic Lung Cancer Cells Mediates VEGFR2 Activation in HUVECs

VEGF receptor 2 (VEGFR2) has been described as a target for the angiogenic effects of VEGF in HUVECs [24,25,26]. From there, it was next tested whether the less VEGF-containing CM of JZL184-treated hypoxic tumor cells also led to decreased VEGFR2 phosphorylation in HUVECs. To this end, the effect of CM obtained from vehicle- or JZL184-treated hypoxic tumor cells on the phosphorylation of VEGFR2 in HUVECs was examined.

**Figure 8:**
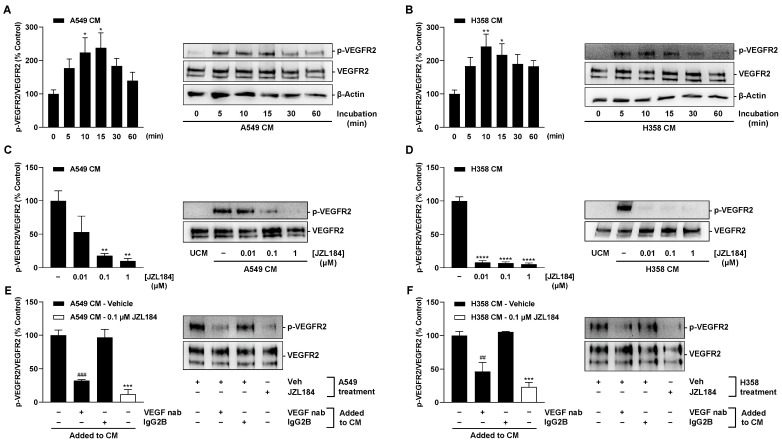
VEGFR2 phosphorylation at tyrosine residue 1175 in HUVECs by CM from hypoxic lung cancer cells: Time-dependent VEGFR2 phosphorylation by CM from vehicle-treated hypoxic A549 (**A**) and H358 cells (**B**); VEGFR2 phosphorylation by CM of previously JZL184-treated hypoxic A549 (**C**) and H358 cells (**D**); VEGFR2 phosphorylation by CM from hypoxic A549 (**E**) and H358 cells (**F**) spiked with a neutralizing VEGF antibody (VEGF nab) or an IgG2B isotype control. HUVECs were incubated with CM of hypoxic A549 or H358 cells for the indicated times (**A**,**B**), 15 min (**C**,**E**), or 10 min (**D**,**F**), followed by VEGFR2 Western blot analysis. CM used for this purpose were obtained after incubation of hypoxic cancer cells with vehicle (**A**,**B**) or vehicle and JZL184 (**C**–**F**) for 48 h. For neutralization studies (**E**,**F**), 1 µg/mL VEGF-A antibody or 1 µg/mL IgG2B isotype control were added to CM from hypoxic vehicle-treated A549 or H358 cells. After incubation at room temperature for 1 h, spiked CM were used for HUVEC incubation. Western blot images are representative of each experiment. Percentage values given refer to VEGFR2 phosphorylation at zero time (**A**,**B**) or to CM of vehicle-treated hypoxic A549 or H358 cells (**C**–**F**) at the above indicated incubation times, each set to 100%. Data represent mean ± SEM of *n* = 7 (**A**,**B**), *n* = 4 (**C**), *n* = 6 (**D**), or *n* = 3 (**E**,**F**). * *p* ≤ 0.05, ** *p* ≤ 0.01, *** *p* ≤ 0.001, **** *p* ≤ 0.0001 vs. zero time (**A**,**B**) or vs. CM from vehicle-treated hypoxic cells (**C**–**F**); ## *p* ≤ 0.01, ### *p* ≤ 0.001 vs. IgG2B isotype control-spiked CM from vehicle-treated hypoxic cells; one-way ANOVA with Dunnett’s (**A**–**D**) or Bonferroni´s (**E**,**F**) post hoc test.

As shown in Figure 8A,B, phosphorylation of VEGFR2 at the tyrosine residue 1175 in HUVECs was upregulated by CM from hypoxic A549 cells, with maximal stimulations achieved after a 15- (CM from A549) or 10-min incubation (CM from H358). Accordingly, the aforementioned incubation times were also used for the experiments shown in Figure 8C–F. Here, the use of CM from cells previously treated with JZL184 resulted in a concentration-dependent decrease in this induction, consistent with the VEGF-lowering effect of the MAGL inhibitor demonstrated before (Figure 6E,F).

Finally, to demonstrate the crucial effect of VEGF released by hypoxic lung cancer cells in VEGFR2 activation of HUVECs, the CM of vehicle-treated tumor cells were spiked with a neutralizing VEGF antibody or an isotype antibody in the reference group. Here, CM containing the neutralizing VEGF antibody significantly inhibited VEGFR2 phosphorylation of HUVECs (Figure 8E,F), again compared with isotype antibody-containing CM. CM of cells treated with 0.1 µM JZL184 in the same experimental set-up were included as controls and again revealed substantial inhibition of VEGFR2 phosphorylation (Figure 8E,F).

### 3.7. The Transcription Factor HIF-1α Mediates Hypoxia-Induced VEGF Expression in H358 but Not in A549 Cells

**Figure 9:**
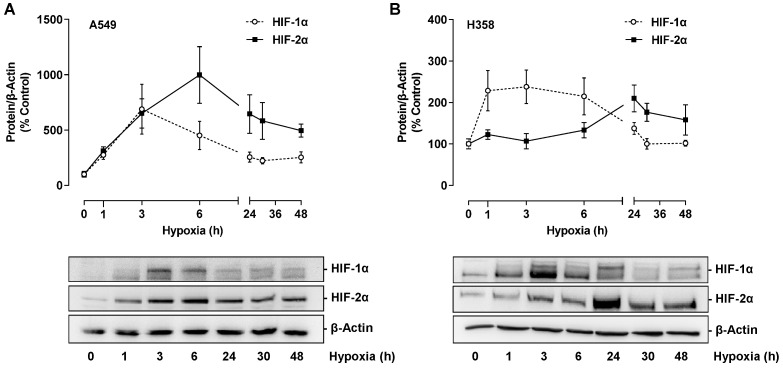
Time-dependent effect of hypoxia on HIF-1α and HIF-2α protein expression in A549 (**A**) or H358 cells (**B**). Cells were incubated under hypoxia for the indicated time periods. Western blot images are representative of each experiment. All percentages given refer to the protein levels determined at zero time, which were set to 100%. Data are mean ± SEM of *n* = 3 (**A**) or *n* = 4 (**B**).

In further experiments, the mechanism of hypoxia-induced VEGF expression should be investigated with regard to possible involvement of HIF transcription factors. For this purpose, the protein expression of HIF-1α and HIF-2α was first analyzed, showing a time-dependent induction of both factors under hypoxia conditions (Figure 9A,B). In A549, levels of both factors increased approximately three-fold within 1 h and reached their maximum after 3 h (HIF-1α) and 6 h (HIF-2α), respectively (Figure 9A). An equally early HIF-1α induction could be detected in H358 cells, whereas maximum HIF-2α concentrations were only evident after 24 h of incubation (Figure 9B).

**Figure 10:**
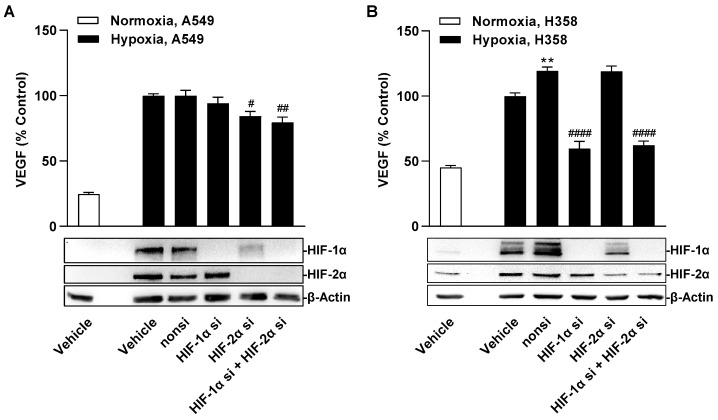
Effect of specific siRNA against HIF-1α and HIF-2α, alone or in combination, on hypoxia-induced VEGF secretion in A549 (**A**) or H358 cells (**B**). Cells were transfected with either non-silencing siRNA (nonsi), HIF-1α- or HIF-2α siRNA for 24 h. Cells were then washed and incubated in serum-free DMEM. After incubation for 48 h, CM were collected and VEGF protein concentrations were determined by ELISA. Monitoring of HIF-1α and HIF-2α protein knockdown was confirmed in parallel with lysates from hypoxic A549 and H358 cells by Western blots after 3 h, 24 h, and 48 h of hypoxia, with the Western blot images shown being representative of the 3 h time point. All percentage values given refer to vehicle-treated hypoxic A549 or H358 cells, each set to 100%. A vehicle-treated normoxic cell group was included to define the hypoxia effect. Data represent mean ± SEM of *n* = 12. ** *p* ≤ 0.01 vs. vehicle-treated hypoxic cells; # *p* ≤ 0.05, ## *p* ≤ 0.01, #### *p* ≤ 0.0001 vs. non-silencing siRNA-treated hypoxic cells; one-way ANOVA with Bonferroni´s post hoc test.

When the expression of both factors was downregulated by specific siRNA, differential regulation of hypoxia-induced VEGF formation was observed in A549 and H358 cells (Figure 10A,B). Thus, knockdown of HIF-1α resulted in no change in VEGF protein levels in the supernatants of A549 cells but in a profound reduction in those of H358 cells. In contrast, knockdown of HIF-2α showed no (H358) or little (A549) inhibitory effect on VEGF secretion. The effects of each group of the siRNA experiment on the viability of hypoxic A549 and H359 cells, respectively, determined under comparable incubation conditions are shown in Supplementary Table S3.

**Figure 11:**
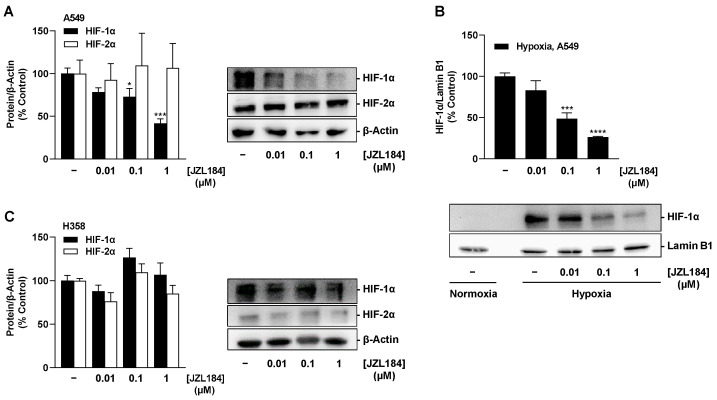
Effect of JZL184 on hypoxia-induced HIF-1α and HIF-2α protein expression in A549 (**A**) or H358 cells (**C**) or in nuclear extracts of A549 cells (**B**). Under hypoxic conditions, A549 and H358 cells were incubated with vehicle or JZL184 for 6 h. Western blot images (**A**–**C**) are representative of each experiment. Percentage values given (**A**–**C**) refer to vehicle-treated hypoxic A549 or H358 cells, each set to 100%. Data represent mean ± SEM of *n* = 4 (**A**,**B**) or *n* = 3 (**C**). * *p* ≤ 0.05, *** *p* ≤ 0.001, **** *p* ≤ 0.0001 vs. vehicle-treated hypoxic cells; one-way ANOVA with Dunnett´s post hoc test.

Different effects were also registered for JZL184 with respect to affecting hypoxia-induced expression of HIF-1α and HIF-2α in both cell lines. Accordingly, JZL184 induced a concentration-dependent partial decrease in HIF-1α protein in hypoxic A549 cells (Figure 11A), which, however, was significant only at 0.1 and 1 µM and did not correspond to the characteristic pattern of all-or-nothing inhibition of VEGF secretion occurring at JZL184 concentrations as low as 0.01 µM (Figure 6E). In addition, a concentration dependence was registered in the analysis of the nuclear extracts (Figure 11B), but again without a corresponding significant inhibitory effect by the JZL184 concentration of 0.01 µM, which, on the other hand effectively reduced VEGF (Figure 6E). Due to the short half-life of HIF-1α as a result of proteasomal degradation, the proteasome inhibitor bortezomib was used as a HIF-1α stabilizer in the nuclear isolation experiments. The efficiency of the nuclear extraction method is shown in Supplementary Figure S6.

In H358 cells, however, no significant inhibitory effect of JZL184 on HIF-1α could be detected, apart from an approximate 12% reduction by the lowest tested concentration of 0.01 µM (Figure 11C). Similarly, no clear effect of JZL184 was detected at the level of HIF-2α expression, apart from a non-significant inhibition of approximately 24% by 0.01 µM JZL184 (Figure 11C).

### 3.8. JZL184 Does Not Inhibit Recombinant VEGF-Induced VEGFR2 Activation and Proliferation of HUVECs

**Figure 12:**
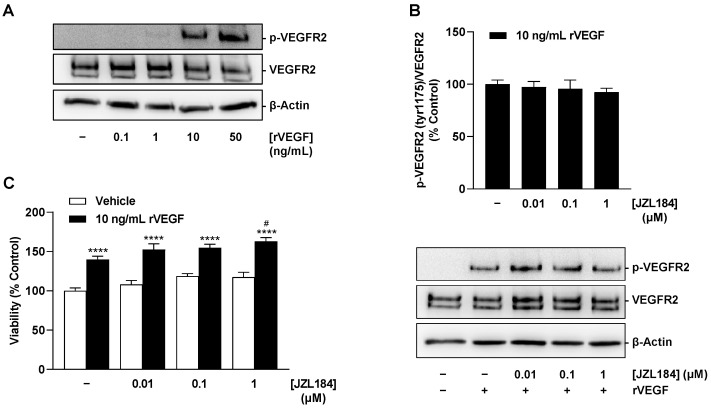
Concentration-dependent effect of rVEGF-165 on VEGFR2 phosphorylation at residue 1175 (**A**), effect of JZL184 on rVEGF-induced VEGFR2 phosphorylation (**B**) and viability (**C**) of HUVECs. In (**A**), HUVECs were stimulated with rVEGF at the indicated concentrations for 5 min. In (**B**), HUVECs were pre-incubated with JZL184 at selected concentrations for 6 h, followed by stimulation with recombinant VEGF-165 (10 ng/mL) for 5 min. In (**C**), viability was determined using the WST-1 assay after 48 h of co-incubation of rVEGF-165 and JZL184. Western blot images are representative of each experiment. All percentages shown refer to vehicle-treated HUVECs set at 100%. Data are mean ± SEM of *n* = 4 (**B**) or *n* = 7–8 (**C**). In (**A**), a representative blot of a total of 2 experiments performed is shown. **** *p* ≤ 0.0001 vs. vehicle-treated HUVECs; # *p* ≤ 0.05 vs. rVEGF-165-stimulated HUVECs; one-way ANOVA with Bonferroni´s post hoc test (**C**). In (**B**), a significant effect of JZL184 was not identified as determined by one-way ANOVA with Dunnett´s post hoc test.

Finally, after conclusively demonstrating that JZL184 impairs VEGF synthesis in tumor cells and subsequently inhibits VEGFR2 activation of HUVECs, the aim was to investigate whether JZL184 also impairs the receptor-activating and proliferative effects of VEGF already present or just released. For this purpose, HUVECs stimulated with recombinant VEGF-165 (rVEGF) were used. In our hands, rVEGF resulted in substantial induction of VEGFR2 phosphorylation at tyrosine residue 1175 even after a 5 min incubation (Figure 12A). However, JZL184 showed no effect on VEGF-induced VEGFR2 activation (Figure 12B) or on enhanced proliferation (Figure 12C) of HUVECs under rVEGF incubation. Thus, in agreement with the previously described control experiments (Supplementary Figure S1), the effect of JZL184 in the selected system appears to be limited to the impairment of VEGF synthesis.

## 4. Discussion

The present study is the first to demonstrate antiangiogenic properties of a MAGL inhibitor, and thus degradation inhibitor of the endocannabinoid 2-AG, under hypoxic conditions due to decreased expression of VEGF in lung cancer cells and subsequently diminished activation of VEGFR2 in human endothelial cells. 

There are several results that support this assumption. Thus, CM derived from hypoxic A549 and H358 lung cancer cells treated with the MAGL inhibitors JZL184 and MJN110 inhibited HUVEC migration and tube formation, two crucial steps in angiogenesis. Involvement of the endocannabinoid system in the effects shown is supported by a selectively significant increase in MAGL substrate 2-AG in lung cancer cells upon incubation with MAGL inhibitors. In line with this finding, the inhibitory effect of CM from hypoxic A549 and H358 cancer cells treated with JZL184 on endothelial cell tube formation and migration potential was partially abolished by antagonists at the CB_1_ and CB_2_ receptors. Inhibition of VEGF secretion was confirmed in the presence of two structurally distinct MAGL inhibitors and, in the case of JZL184, was partially abolished by CB_1_ and CB_2_ antagonists, consistent with the data on angiogenesis. Finally, altered endothelial VEGF signaling resulting from VEGF downregulation triggered by JZL184 in hypoxic lung cancer cells was confirmed by analyzing the effect of CM of cancer cells on VEGFR2 phosphorylation of HUVECs. Here, VEGFR2 phosphorylation was significantly inhibited by CM of A549 or H358 cells previously treated with JZL184 compared with CM of vehicle-treated cells. On the other hand, JZL184 showed no effect on VEGFR2 activation induced by exogenous rVEGF, ruling out a direct intervention of JZL184 in VEGFR signaling in HUVECs.

Antiangiogenic properties of CB_1_ and CB_2_ receptor agonists have been reported before in vitro and in vivo [27,28,29,30,31,32]. However, corresponding evidence on MAGL inhibitors is limited. In one investigation, the MAGL inhibitor URB602 reduced the number of microvessels in a mouse colonic xenograft, suggesting an involvement of antiangiogenic effects in the tumor regression induced here by this compound [12]. In another recent study performed on normoxic lung cancer cells, angiogenic parameters of HUVECs were reduced by CM of lung cancer cells previously incubated with MAGL inhibitors, with the release of antiangiogenic TIMP-1 playing a critical role in this process [13].

In the present work, multiplex technology was used to analyze 10 different factors involved in angiogenesis. This revealed a selective inhibition of VEGF formation by JZL184, which was confirmed by ELISA and also detected at the mRNA level by RT-qPCR. As shown by our data, inhibition of VEGF expression seems to be an important factor for the VEGF-lowering effect of JZL184, which completely inhibited hypoxia-induced VEGF mRNA and protein formation at a concentration as low as 0.01 µM. In agreement with our results, reduced VEGF expression was also demonstrated after in vivo administration of a MAGL inhibitor in mouse colon carcinoma xenografts [12]. Moreover, activation of CB_1_ [29] and CB_2_ receptors [30] has been shown to reduce the expression of VEGF in tumors, accompanied by an antiangiogenic effect of the corresponding agonists.

In our hands, VEGF inhibition was not due to cytotoxic effects of JZL184, which even under hypoxic conditions did not induce a decrease in viability of the lung cancer cells used. Consistent with this, JZL184 at a concentration range of 0.01 to 10 µM also did not significantly reduce viability or proliferation in a study we performed on normoxic A549 cells under serum-containing and serum-free conditions [4]. Thereby, JZL184 likewise elicited no significant change in colony-forming properties of A549 cells [4]. The effect of MAGL inhibitors on tumor cell viability was recently reviewed [1] and appears to depend on the particular experimental conditions, the cell line used, the duration of the effect, and the concentration used.

According to a number of reports, VEGF and its membrane receptor VEGFR2 play an important role in regulating physiological and pathophysiological angiogenesis (for review, see [11]). Bevacizumab, a monoclonal antibody that binds soluble VEGF, thereby preventing its binding to receptors, was therefore developed as the first pharmacotherapy to combat tumor angiogenesis. A small molecule strategy subsequently launched with sunitinib and sorafinib directly targets VEGFR2 activation by tyrosine kinase inhibition (for review, see [33]). Remarkably, in the HUVECs we also used, MIL60, a VEGF-neutralizing antibody, inhibited VEGF-triggered proliferation, migration, and tube formation via the VEGFR2 pathway [26]. In our control experiments, VEGFR2 activation induced by CM from vehicle-treated hypoxic lung cancer cells was inhibited by a neutralizing antibody against VEGF as well as when CM from hypoxic lung cancer cells treated with VEGF-lowering JZL184 were used. Surprisingly, the VEGF-neutralizing antibody did not completely inhibit the phosphorylation of VEGFR2 in HUVECs, which may be due to the fact that other factors, such as IL-8, which was also detectable in the CM, can lead to transactivation of VEGFR2 [34] but are not inhibited by the neutralizing antibody.

As expected, hypoxic treatment of A549 and H358 cells resulted in a time-dependent increase in HIF-1α and HIF-2α protein levels. These results confirm the known stabilization of both HIF-α subunits under hypoxia, which prevents the modification by HIF-specific prolyl hydroxylases and subsequent ubiquitination and proteasomal degradation that otherwise occurs under normoxic conditions (for review, see [35]). After accumulation in the cytoplasm, HIF-α enters the nucleus and dimerizes with HIF-β to form the HIF complex, which acts as a transcription factor for hundreds of genes, including those involved in angiogenesis such as VEGF, platelet-derived growth factor (PDGF) and angiopoietin-1 (for review, see [36]). The efficient accumulation of HIF-1 in the nucleus required for potential transcriptional modulation was convincingly demonstrated in this work with the hypoxia-induced HIF-1α increase in nuclear fractions of A549 cells as well as with the concentration-dependent inhibition by JZL184 also detected there. Downregulation of HIF-1α under hypoxic conditions has also been previously described in glioma cells exposed to cannabidiol [37] and in colon cancer xenografts from mice treated with the hexahydrocannabinol analog LYR-8 [38].

Regulation of VEGF under hypoxic conditions has been described for HIF-1α [39,40,41] and HIF-2α [42,43,44]. However, the siRNA experiments we performed do not indicate a major role for both HIF-1α and HIF-2α in hypoxia-induced VEGF secretion in A549 cells. On the other hand, HIF-1α knockdown was associated with a strong suppression of VEGF release from hypoxic H358 cells. These divergent cell type-dependent findings are consistent with the literature, which repeatedly reported HIF-1-independent VEGF inductions under hypoxia conditions in addition to HIF-1-dependent ones (for review, see [45]). For example, in hypoxic colon cancer cells, activation of PI3K/Rho/ROCK and c-Myc have been revealed to be alternative pathways of appropriate VEGF induction [46]. In another work, the flavonoids fisetin, luteolin, galangin, and quercetin were shown to inhibit hypoxia-induced VEGF formation in the lung cancer squamous cell carcinoma cell line NCI-H157, but concomitantly caused induction (rather than inhibition) of HIF-1α expression [47].

Independent of the finding of HIF-1α-independent VEGF regulation in hypoxic A549 cells, the concentration-dependent decrease in HIF-1α protein in A549 whole cell lysates as well as in nuclear fractions triggered by JZL184 opens new perspectives and research approaches for this substance. Indeed, increased protein levels of HIF-1α and HIF-2α are associated with poor prognosis and metastasis in a number of cancers, including lung cancer (for review, see [48]). In particular, HIF-1α activates the transcription of genes involved in virtually all aspects of tumor biology, inducing, for example, invasion and metastasis as well as resistance to radiation and chemotherapy, in addition to angiogenesis. Therefore, HIF inhibition represents a potential target that has been and is being investigated in preclinical and clinical studies (for review, see [49,50,51,52]) with mechanisms of action such as inhibition of HIF-1α protein synthesis and transcriptional activity or induction of HIF-1α hydroxylation and subsequent ubiquitination and proteasomal degradation. In light of this, the corresponding mechanism of JZL184 in terms of the HIF-1α decrease shown, as well as its functional consequence, should be addressed in future studies.

Another issue to be clarified in the present work was the possible effect of the MAGL inhibitor JZL184 via the reduction in free fatty acids. Thus, the experiments we performed with receptor antagonists suggested that only part of the antiangiogenic and VEGF-lowering effect of JZL184 proceeds via cannabinoid receptors. To address a corresponding mechanism, add-back experiments with palmitic acid have been repeatedly described in the literature. These found an involvement of fatty acid reduction by JZL184 in its antimigrative effect on prostate cancer cells [2] as well as on MAGL-overexpressing melanoma and ovarian cancer cells [8], but not in its anti-invasive effect on lung cancer cells [4] or its antiangiogenic impact on the microenvironment of normoxic lung cancer cells [13]. Consistent with the latter report, in the present study, the addition of palmitic acid also failed to restore the JZL184-induced decreased VEGF formation in hypoxic A549 and H358 cells as well as the decreased angiogenic properties of HUVECs upon contact with CM from JZL184-treated cancer cells. However, it is difficult to make a conclusive statement because many aspects of lipid metabolism are modulated under hypoxia (for review, see [53,54]). Thus, hypoxia-associated HIF-1 activation in various cancer cells leads to the downregulation of lipid droplet degradation mediated by adipose triglyceride lipase (ATGL), the key enzyme for intracellular lipolysis [55]. Accordingly, a 24 h hypoxic treatment of cancer cells here resulted in a 2.5- to 3-fold lower release of free fatty acids compared to normoxic conditions [55]. Under these circumstances, pharmacological suppression of glyceride degradation (e.g., by MAGL inhibitors) is likely to be superimposed by the hypoxia effect, making it difficult to draw a clear conclusion about the drug mechanism. In addition, the regulation of MAGL by hypoxia or HIF-1 is currently unclear [54].

It should also be added that in the present study, we were unable to register clear concentration dependencies in the effect of JZL184 in many cases, with maximum effects already occurring at the lowest concentration of 0.01 µM used. In this context, however, it should be noted that JZL184 inhibits the MAGL enzyme highly potently with IC_50_ values in the nanomolar range [14]. Accordingly, in a previous study by our group, JZL184 already elicited a highly significant induction of 2-AG synthesis in normoxic A549 cells at a concentration as low as 0.01 µM [4].

Collectively, the present study is the first to elucidate an antiangiogenic mechanism of MAGL inhibitors under hypoxia essential for neoangiogenesis in malignant tumors. Therefore, another necessary step was taken in the preclinical characterization of the anticancer activity of MAGL inhibitors.

## Data Availability

Data are available upon reasonable request from the first author.

## Supplementary Materials

The following supplementary information can be downloaded at https://www.mdpi.com/article/10.3390/cells12192332/s1, Supplementary Figure S1. Effect of JZL184 on migration (left), tube formation (middle), and viability (right) of HUVECs. Supplementary Figure S2. Effect of CM of vehicle- and JZL184-treated hypoxic A549 (**A**) and H358 cells (**B**) on the viability of HUVECs under the experimental conditions of tube formation assay. Supplementary Figure S3. Effect of MJN110 (**A**–**C**) or CM collected from MJN110-treated hypoxic A549 (**D**–**F**) or H358 cells (**G**–**I**) on migration, tube formation, and viability of HUVECs, and impact of MJN110 on endocannabinoid concentrations in A549 cells (**J**) and VEGF secretion by A549 (**K**) and H398 cells (**L**). Supplementary Figure S4. Effect of CM of hypoxic A549 (**A**,**B**) and H358 cells (**C**,**D**) treated with AM-251 (CB_1_ receptor antagonist), AM-630 (CB_2_ receptor antagonist), and capsazepine (CPZ, TRPV1 antagonist) on angiogenic properties of HUVECs and influence of the above antagonists on VEGF release by hypoxic A549 (**E**) and H358 cells (**F**). Supplementary Figure S5. Effect of JZL184 on the release of IL-6 (**A**), IL-8 (**B**), or TNFα (**C**) from hypoxic A549 cells. Supplementary Figure S6. The efficiency of nuclear isolation and the effect of the proteasome inhibitor bortezomib on HIF-1α stabilization. Supplementary Table S1. Parameters of the interface of the LCMS-8050. Supplementary Table S2. Precursor and product ions of the measured endocannabinoids and internal standards with their respective ionization parameters. Supplementary Table S3. Effect of the different test substances or transfections on the viability of A549 and H358 cells in MTT assay. Supplementary Table S4. Effect of palmitic acid alone or in combination with JZL184 on the viability of hypoxic A549 and H358 cells in crystal violet assay.
