# Supplementary material for: Antiangiogenic Action of JZL184 on Endothelial Cells via Inhibition of VEGF Expression in Hypoxic Lung Cancer Cells
Source: Cells. 2023 Sep 22;12(19):2332. doi: 10.3390/cells12192332 (PMC10572003; doi:10.3390/cells12192332)
Supplement: Supplementary file 1 [file cells-12-02332-s001.zip › cells-2521481-supplementary.pdf]

cells

MDPI

Supplementary Material

Antiangiogenic Action of JZL184 on Endothelial Cells via Inhibition of VEGF Expression in Hypoxic Lung Cancer Cells

Felix Wittig [1], Liza Pannenberg [1], Rico Schwarz [1], Sander Bekeschus [2], Robert Ramer [1], Burkhard Hinz [1,*]

[1] Institute of Pharmacology and Toxicology, Rostock University Medical Center, Schillingallee 70, 18057 Rostock, Germany; felix.wittig@med.uni-rostock.de (F.W.); liza.pannenberg@gmail.com (L.P.); rschwarz-arbeit@gmx.de (R.S.); robert.ramer@med.uni-rostock.de (R.R.)

[2] ZIK *plasmatis*, Leibniz Institute for Plasma Science and Technology (INP), Felix-Hausdorff-Str. 2, 17489 Greifswald, Germany; sander.bekeschus@inp-greifswald.de

* Correspondence: burkhard.hinz@med.uni-rostock.de; Tel.: +49-381-494-5770

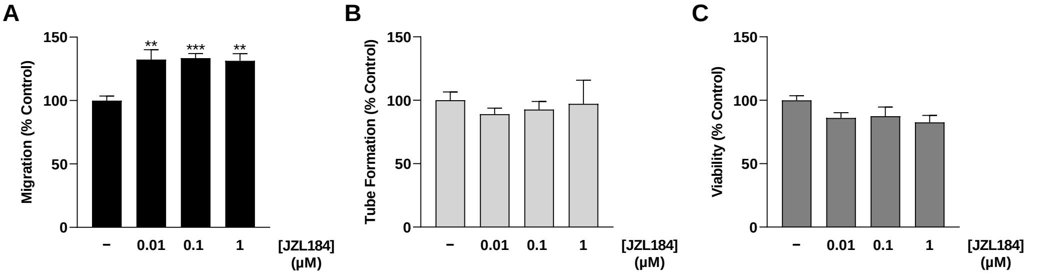

Supplementary Figure S1. Effect of JZL184 on migration (**A**), tube formation (**B**), and viability (**C**) of HUVECs. HUVECs were incubated directly with vehicle or JZL184. Angiogenic features were determined after incubation of HUVECs with vehicle or JZL184 for 6 h (tube formation analysis) or 24 h (migration and viability assay). Percentages shown refer to vehicle-treated HUVECs, each set to 100%. Data represent mean ± SEM of $n = 6$ (left, middle) or $n = 8$ (right). ** $p \leq 0.01$, *** $p \leq 0.001$ vs. vehicle; one-way ANOVA with Dunnett´s post hoc test.

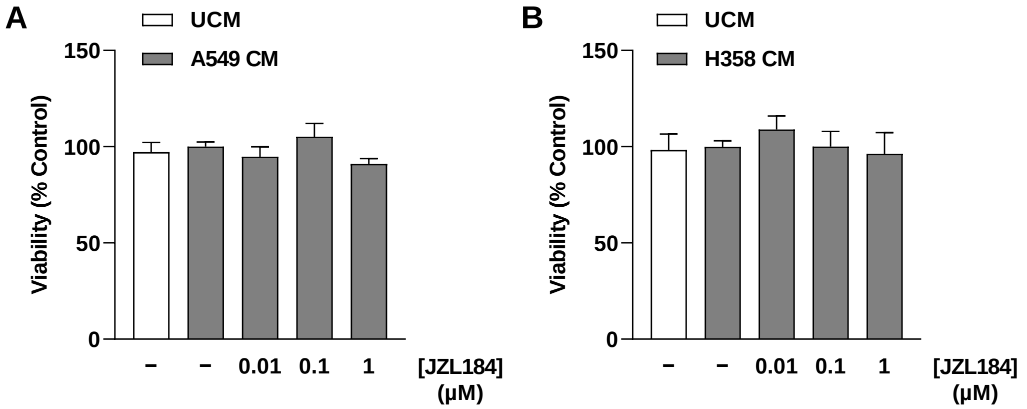

Supplementary Figure S2. Effect of CM of vehicle- and JZL184-treated hypoxic A549 (**A**) and H358 cells (**B**) on the viability of HUVECs under the experimental conditions of tube formation assay. Hypoxic A549 and H358 cells, respectively, were first treated with vehicle or JZL184 for 48 h, followed by the collection of CM, which were then used to suspend HUVECs on 48-well plates coated with Matrigel. After an incubation period of 6 h, a WST-1 assay was performed. Percentages shown refer to HUVECs treated with CM derived from vehicle-treated hypoxic cancer cells, set to 100%. Serum-free DMEM (unconditioned medium, UCM) was included to define a potential hypoxia effect. Data represent mean ± SEM of $n = 6$. A significant effect of JZL184 was excluded by one-way ANOVA with Dunnett´s post hoc test.

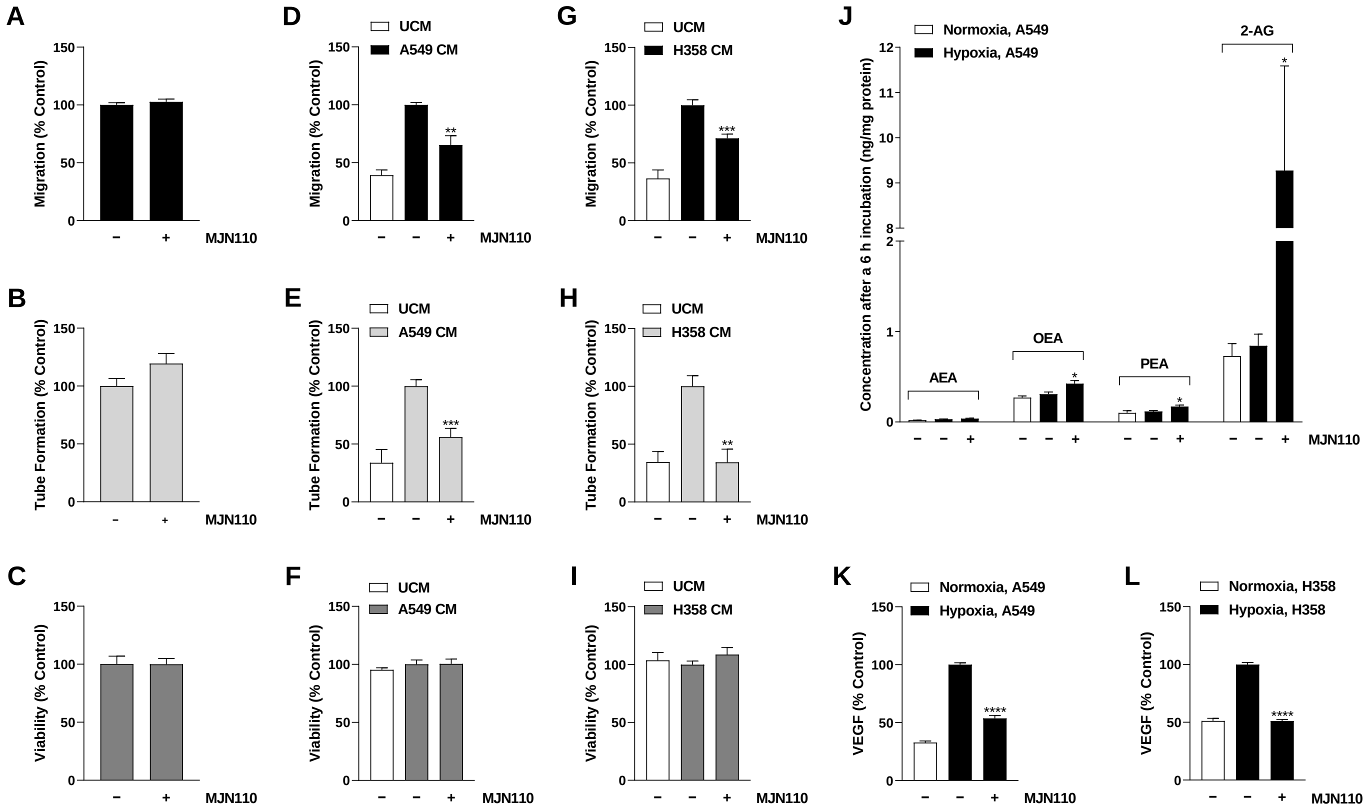

Supplementary Figure S3. Effect of MJN110 (**A**–**C**) or CM collected from MJN110-treated hypoxic A549 (**D**–**F**) or H358 cells (**G**–**I**) on migration, tube formation, and viability of HUVECs, and impact of MJN110 on endocannabinoid concentrations in A549 cells (**J**) and VEGF secretion by A549 (**K**) and H398 cells (**L**). In (**A**–**C**), HUVECs were directly treated with vehicle or MJN110 (0.1 µM). In contrast, in (**D**–**I**), HUVECs were suspended in CM. The CM used for this purpose was taken from hypoxic A549 (**D**–**F**) or H358 cells (**G**–**I**), respectively, treated with vehicle or MJN110 (0.1 µM) for 48 h. Angiogenic features were determined after incubation of HUVECs with vehicle or MJN110 (**A**–**C**) or CM from vehicle or MJN110-treated cancer cells (**D**–**I**) for 6 h (tube formation analysis) or 24 h (migration and viability assay). To determine the impact of MJN110 on cellular endocannabinoid levels (**J**) or VEGF secretion (**K**,**L**), cancer cells were incubated for 6 h (**J**) or 48 h (**K**,**L**) with vehicle or MJN110 (0.1 µM) under hypoxic conditions. Percentages given refer to vehicle-treated HUVECs (**A**–**C**), HUVECs treated with CM derived from vehicle-treated hypoxic cancer cells (**D**–**I**), or vehicle-treated hypoxic A549 or H358 cells (**K**,**L**), each set to 100%. In (**J**), mean concentrations are shown. Serum-free DMEM (unconditioned medium, UCM, **D**–**I**) or vehicle-treated normoxic cancer cells (**J**–**L**) were included to define the hypoxia effect. Data represent mean ± SEM of $n = 6$ (**A**,**B**,**D**,**E**,**G**,**H**), $n = 8$ (**C**,**F**,**I**,**K**,**L**), or $n = 4$ (**J**). * $p \leq 0.05$, ** $p \leq 0.01$, *** $p \leq 0.001$, **** $p \leq 0.0001$ vs. vehicle (**A**–**C**), CM from vehicle-treated hypoxic cancer cells (**D**–**I**) or vehicle-treated hypoxic cancer cells (**J**–**L**); Student's unpaired two-tailed *t*-test.

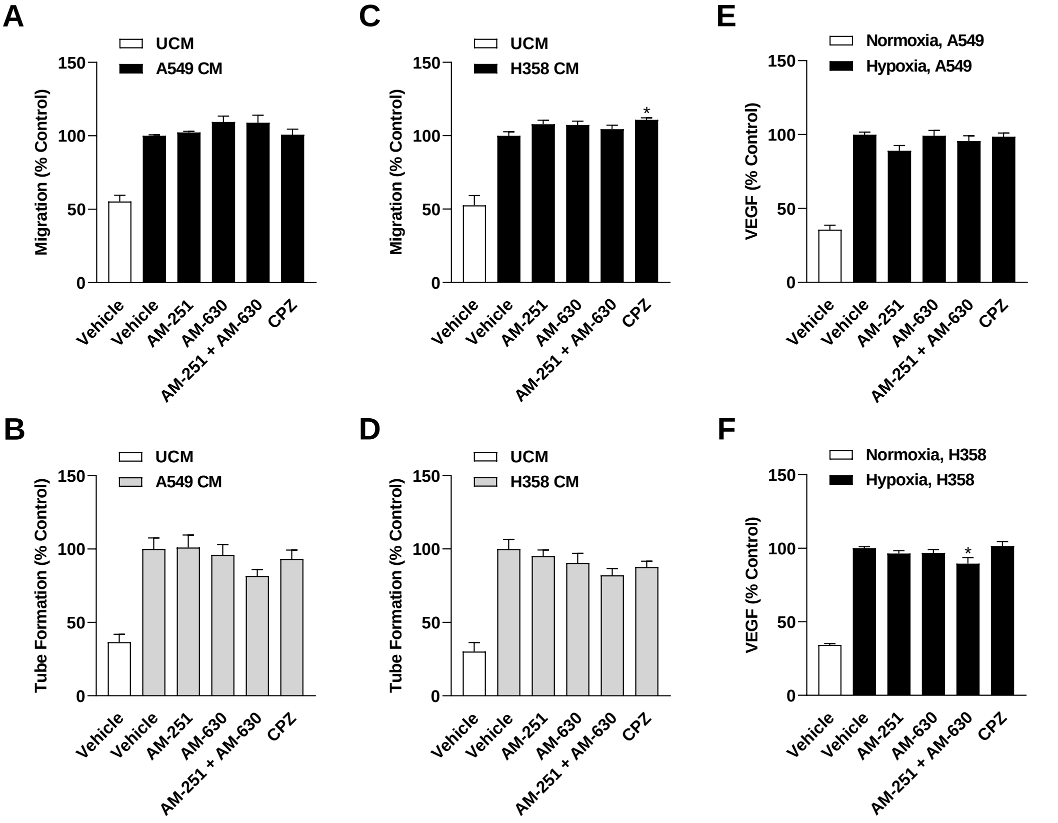

Supplementary Figure S4. Effect of CM of hypoxic A549 (**A**,**B**) and H358 cells (**C**,**D**) treated with AM-251 (CB_1 receptor antagonist), AM-630 (CB_2 receptor antagonist), and capsazepine (CPZ, TRPV1 antagonist) on angiogenic properties of HUVECs and influence of the above antagonists on VEGF release by hypoxic A549 (**E**) and H358 cells (**F**). Cells were pre-incubated with the respective receptor antagonist (all at a final concentration of 1 μM) or its vehicle for 30 min under normoxic conditions, followed by equilibration and incubation under hypoxia for 48 h. Thereafter, CM were used to determine its effects on HUVEC migration (**A**,**C**) and tube formation (**B**,**D**) or to assess VEGF levels by ELISA (**E**,**F**). Migration and tube formation of HUVECs were determined after incubation with CM of cancer cells for 24 h (migration) or 6 h (tube formation analysis). Percentage values refer to HUVECs treated with CM derived from vehicle-treated hypoxic cancer cells (**A**–**D**) or to vehicle-treated hypoxic A549 or H358 cells (**E**,**F**), each set to 100%. Serum-free DMEM (unconditioned medium, UCM; **A**–**D**) or a vehicle-treated normoxic cell group (**E**,**F**) was included to define the hypoxia effect. Data represent mean ± SEM of $n = 6$ (**A**–**D**), $n = 12$ (**E**), or $n = 8$ (**F**). * $p \leq 0.05$ vs. vehicle-treated hypoxic cells; one-way ANOVA with Dunnett´s post hoc test.

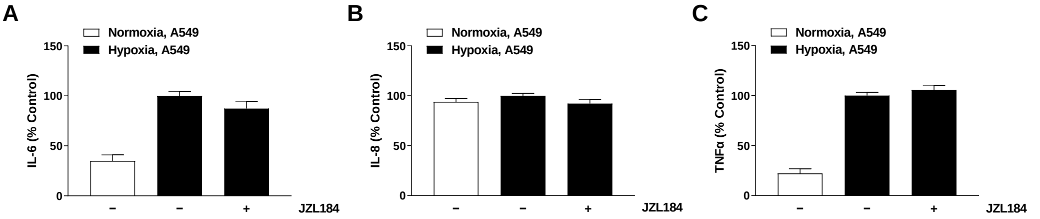

Supplementary Figure S5. Effect of JZL184 on the release of IL-6 (**A**), IL-8 (**B**), or TNFα (**C**) from hypoxic A549 cells. The CM used were from hypoxic A549 cells previously incubated for 48 h with vehicle or JZL184 (0.1 μM). A vehicle-treated normoxic cell group was included to show a potential hypoxia effect. Angiogenic mediators were determined in cell culture supernatants using ELISA. Because of low TNFα secretion, 2 mL of each CM was concentrated to a final volume of 100 μL before measurement. All percentage values refer to vehicle-treated hypoxic A549 cells, each set to 100%. Data represent mean ± SEM of $n = 10$–11 (**A**), $n = 10$–12 (**B**), or $n = 6$ (**C**). Significant differences between groups were not identified as determined by Student's unpaired two-tailed *t*-test.

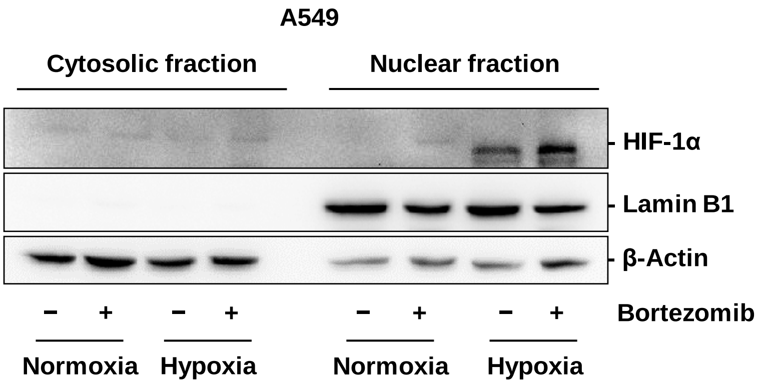

Supplementary Figure S6. The efficiency of nuclear isolation and the effect of the proteasome inhibitor bortezomib on HIF-1α stabilization. A549 cells treated with vehicle in serum-free DMEM for 6 h under normoxic or hypoxic conditions were then harvested followed by isolation of the cytoplasmic and nuclear fractions. As indicated, samples were processed in the absence or presence of bortezomib. In the latter case, bortezomib was added as a HIF-1α stabilizer to each component used for cell harvesting and to the solubilization buffers at a final concentration of 1 or 10 μM, as described in Section 2.9. The cytoplasmic and nuclear fractions were analyzed for HIF-1α and housekeeping proteins (lamin B1 for the nuclear fraction, β-actin for the cytoplasmic fraction) by Western blot analysis.

Supplementary Table S1. Parameters of the interface of the LCMS-8050.

Interface ESI parameter	Value
Nebulizing gas flow	2 L/min
Heating gas flow	10 L/min
Interface temperature	300 °C
Desolvation temperature	526 °C
DL temperature	200 °C
Heat block temperature	400 °C
Dry gas flow	10 L/min

Supplementary Table S2. Precursor and product ions of the measured endocannabinoids and internal standards with their respective ionization parameters.

Substance	Precursor (*m*/*z*)	Product (*m*/*z*)	Dwell time (msec)	Q1 Pre Bias (V)	CE	Q3 Pre Bias (V)
AEA	348.10	62.15	15	−14	-17	-25
	348.10	91.10	15	-11	-40	-18
	348.10	44.10	15	-18	-33	-18
2-AG	379.30	287.15	15	-14	-16	-21
	379.30	269.20	15	-15	-18	-20
	379.30	91.15	15	-15	-54	-18
OEA	326.20	62.15	15	-24	-16	-25
	326.20	43.85	15	-17	-37	-16
	326.20	54.90	15	-10	-42	-26
PEA	300.40	62.00	15	-13	-16	-12
	300.40	44.50	15	-11	-29	-16
	300.40	283.15	15	-11	-10	-15
AEA-d8	356.20	63.15	15	-11	-22	-30
	356.20	44.55	15	-13	-54	-25
	356.20	64.15	15	-11	-15	-11
2-AG-d5	384.20	287.20	15	-15	-18	-30
	384.20	269.10	15	-15	-17	-19
	384.20	91.15	15	-12	-48	-10

Supplementary Table S3. Effect of the different test substances or transfections on the viability of hypoxic A549 and H358 cells in MTT assay. A549 and H358 cells were processed and treated in 48-well plates as described in Section 2.7. The incubation schemes of the individual experiments (**A**–**F**) correspond to the setups described in detail in other Figure legends (reference in column 2 of Table S3) and include the incubation times used there until CM were obtained. Hypoxic cancer cells treated with vehicle were set to 100%. Data represent mean ± SEM of $n = 12$ (**A**,**B**,**E**) or $n = 8$ (**C**,**D**,**F**). * $p \leq 0.05$, ** $p \leq 0.01$, **** $p \leq 0.0001$ vs. respective vehicle; # $p \leq 0.05$, ## $p \leq 0.01$, ### $p \leq 0.001$ vs. JZL184-treated hypoxic cells; one-way ANOVA with Dunnett´s post hoc test (**A**,**D**), Student's unpaired two-tailed *t*-test (**B**), or one-way ANOVA with Bonferroni´s post hoc test (**C**,**E**,**F**).

Exp.	Incubation Scheme Corresponds to	Treatment Groups	Viability A549 cells (%)	Viability H358 cells (%)
**A**	Figure 2	Vehicle	100.0 ± 1.8	100.0 ± 2.3
		JZL184 (0.01 μM)	102.6 ± 3.1	105.9 ± 2.8
		JZL184 (0.1 μM)	102.8 ± 3.5	110.0 ± 3.4
		JZL184 (1 μM)	102.3 ± 2.3	101.3 ± 3.6
**B**	Figure S3D–I	Vehicle	100.0 ± 3.1	100.0 ± 2.5
		MJN110 (0.1 μM)	100.8 ± 2.6	92.9 ± 3.1
**C**	Figure 4A–D	Vehicle	100.0 ± 2.7	100.0 ± 1.7
		JZL184 (0.1 μM)	97.8 ± 5.9	101.4 ± 2.3
		JZL184 (0.1 μM) + AM-251 (1 μM)	104.8 ± 3.9	88.5 ± 4.3 (#)
		JZL184 (0.1 μM) + AM-630 (1 μM)	100.7 ± 5.3	87.9 ± 3.9 (#)
		JZL184 (0.1 μM) + AM-251 (1 μM) + AM-630 (1 μM)	97.0 ± 6.0	87.3 ± 2.3 (##)
		JZL184 (0.1 μM) + Capsazepine (1 μM)	94.7 ± 1.9	94.4 ± 2.2
**D**	Figure S4A–D	Vehicle	100.0 ± 3.0	100.0 ± 2.0
		AM-251 (1 μM)	109.2 ± 5.0	100.7 ± 0.9
		AM-630 (1 μM)	92.0 ± 5.4	86.1 ± 5.2 (**)
		AM-251 (1 μM) + AM-630 (1 μM)	100.0 ± 5.3	95.6 ± 2.9
		Capsazepine (1 μM)	95.5 ± 3.5	98.8 ± 1.5
**E**	Figure 4E–H	Vehicle	100.0 ± 2.4	100.0 ± 1.1
		JZL184 (0.1 μM)	98.8 ± 1.6	99.3 ± 2.5
		JZL184 (0.1 μM) + Palmitic Acid (10 μM)	80.8 ± 3.7 (###)	82.6 ± 4.1 (##)
		Palmitic Acid (10 μM)	78.1 ± 3.9 (****)	77.0 ± 4.5 (****)
**F**	Figure 10	Vehicle	100.0 ± 2.6	100.0 ± 2.7
		Nonsilencing siRNA (10 nM)	104.6 ± 3.7	90.2 ± 1.0 (*)
		HIF-1α siRNA (10 nM)	98.9 ± 1.7	82.9 ± 2.2
		HIF-2α siRNA (10 nM)	115.9 ± 4.9	84.9 ± 3.4
		HIF-1α siRNA (10 nM) + HIF-2α siRNA (10 nM)	114.8 ± 4.6	92.8 ± 2.5

Supplementary Table S4. Effect of palmitic acid alone or in combination with JZL184 on the viability of hypoxic A549 and H358 cells in crystal violet assay. A549 and H358 cells were processed and treated in 48-well plates as described in Section 2.7. The incubation schemes of the listed experiments correspond to the setups already presented in detail elsewhere (reference in column 1 of Table S4) and include the incubation times used there until CM were obtained. Hypoxic cancer cells treated with vehicle were set at 100%. Data represent mean ± SEM of n = 8. Significant differences between groups were excluded by one-way ANOVA with Bonferroni´s post hoc test.

Incubation Scheme Corresponds to	Treatment Groups	Viability A549 cells (%)	Viability H358 cells (%)
Figure 4E–H	Vehicle	100.0 ± 1.2	100.0 ± 1.2
	JZL184 (0.1 μM)	97.7 ± 1.8	94.0 ± 2.5
	JZL184 (0.1 μM) + Palmitic Acid (10 μM)	96.9 ± 1.2	95.3 ± 2.5
	Palmitic Acid (10 μM)	96.3 ± 1.1	96.1 ± 2.2
